# Optimizing the learning rate for adaptive estimation of neural encoding models

**DOI:** 10.1371/journal.pcbi.1006168

**Published:** 2018-05-29

**Authors:** Han-Lin Hsieh, Maryam M. Shanechi

**Affiliations:** 1 Ming Hsieh Department of Electrical Engineering, Viterbi School of Engineering, University of Southern California, Los Angeles, California, United States of America; 2 Neuroscience Graduate Program, University of Southern California, Los Angeles, California, United States of America; University College London, UNITED KINGDOM

## Abstract

Closed-loop neurotechnologies often need to adaptively learn an encoding model that relates the neural activity to the brain state, and is used for brain state decoding. The speed and accuracy of adaptive learning algorithms are critically affected by the learning rate, which dictates how fast model parameters are updated based on new observations. Despite the importance of the learning rate, currently an analytical approach for its selection is largely lacking and existing signal processing methods vastly tune it empirically or heuristically. Here, we develop a novel analytical calibration algorithm for optimal selection of the learning rate in adaptive Bayesian filters. We formulate the problem through a fundamental trade-off that learning rate introduces between the steady-state error and the convergence time of the estimated model parameters. We derive explicit functions that predict the effect of learning rate on error and convergence time. Using these functions, our calibration algorithm can keep the steady-state parameter error covariance smaller than a desired upper-bound while minimizing the convergence time, or keep the convergence time faster than a desired value while minimizing the error. We derive the algorithm both for discrete-valued spikes modeled as point processes nonlinearly dependent on the brain state, and for continuous-valued neural recordings modeled as Gaussian processes linearly dependent on the brain state. Using extensive closed-loop simulations, we show that the analytical solution of the calibration algorithm accurately predicts the effect of learning rate on parameter error and convergence time. Moreover, the calibration algorithm allows for fast and accurate learning of the encoding model and for fast convergence of decoding to accurate performance. Finally, larger learning rates result in inaccurate encoding models and decoders, and smaller learning rates delay their convergence. The calibration algorithm provides a novel analytical approach to predictably achieve a desired level of error and convergence time in adaptive learning, with application to closed-loop neurotechnologies and other signal processing domains.

## Author summary

Closed-loop neurotechnologies for treatment of neurological disorders often require adaptively learning an encoding model to relate the neural activity to the brain state and decode this state. Fast and accurate adaptive learning is critically affected by the learning rate, a key variable in any adaptive algorithm. However, existing signal processing algorithms select the learning rate empirically or heuristically due to the lack of a principled approach for learning rate calibration. Here, we develop a novel analytical calibration algorithm to optimally select the learning rate. The learning rate introduces a trade-off between the steady-state error and the convergence time of the estimated model parameters. Our calibration algorithm can keep the steady-state parameter error smaller than a desired value while minimizing the convergence time, or keep the convergence time faster than a desired value while minimizing the error. Using extensive closed-loop simulations, we show that the calibration algorithm allows for fast learning of accurate encoding models, and consequently for fast convergence of decoder performance to high values for both discrete-valued spike recordings and continuous-valued recordings such as local field potentials. The calibration algorithm can achieve a predictable level of speed and accuracy in adaptive learning, with significant implications for neurotechnologies.

## Introduction

Recent technological advances have enabled the real-time recording and processing of different invasive neural signal modalities, including the electrocorticogram (ECoG), local field potentials (LFP), and spiking activity [1]. This real-time recording capability has allowed for the development of various neurotechnologies to treat neurological disorders. For example, motor brain-machine interfaces (BMI) have the potential to restore movement to disabled patients by recording the neural activity—such as ECoG, LFP, or spikes—in real time, decoding from this activity the motor intent of the subject, and using the decoded intent to actuate and control an external device [2–12]. Closed-loop deep brain stimulation (DBS) systems, e.g., for treatment of Parkinson’s disease, use recordings such as ECoG or LFP to decode the underlying diseased state of the brain and adjust the electrical stimulation pattern to an appropriate brain region, e.g., the subthalamic nucleus (STN) [13–16]. These neurotechnologies are examples of closed-loop neural systems.

**Fig 1:**
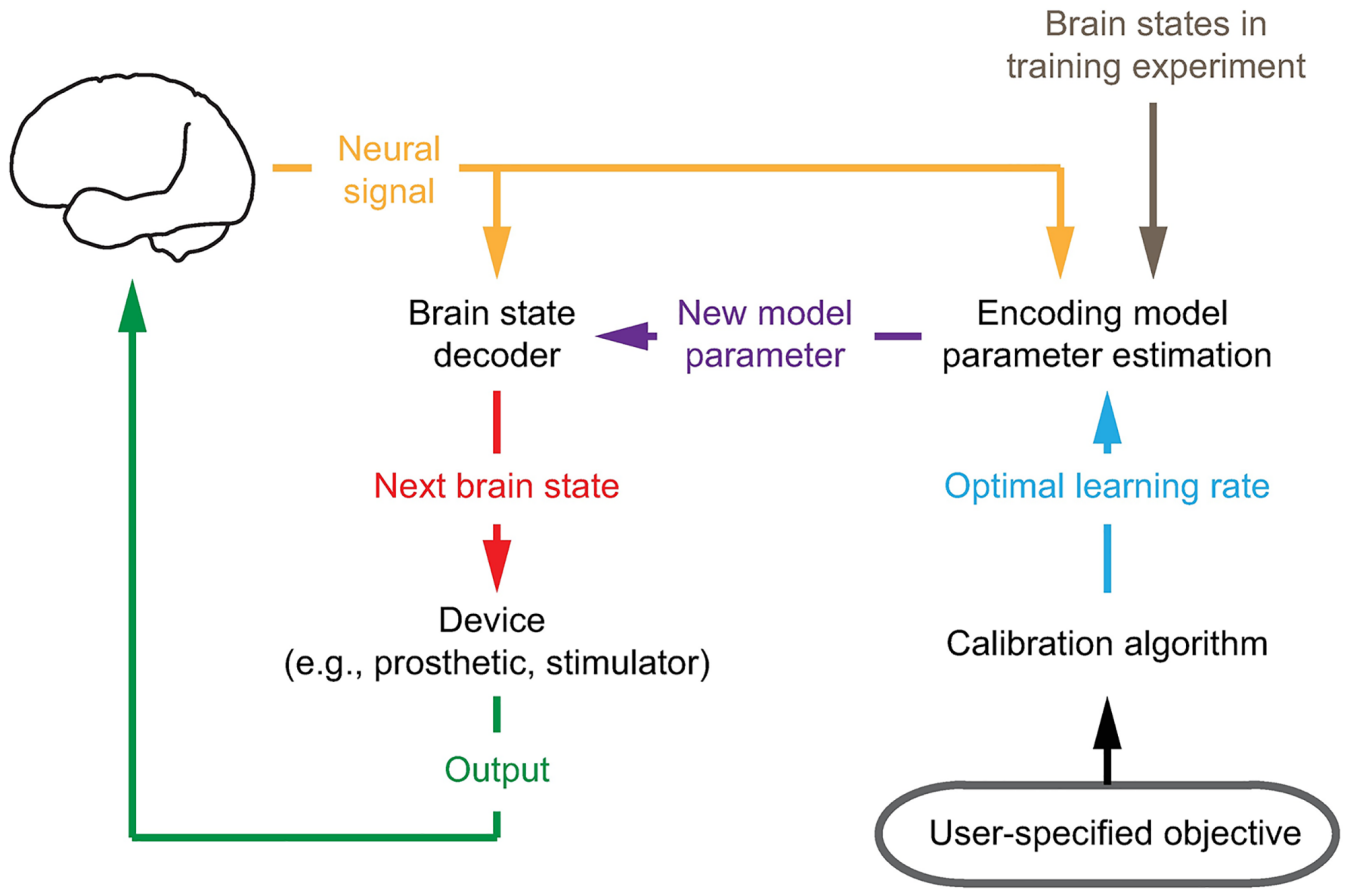
Closed-loop neural system. Closed-loop neural systems often need to learn an encoding model adaptively and in real time. The encoding model describes the relationship between neural recordings and the brain state. For example, the relevant brain state in motor BMIs is the intended velocity and in DBS systems is the disease state, e.g., in Parkinson’s disease. The neural system uses the learned encoding model to decode the brain state. This decoded brain state is then used, for example, to move a prosthetic in motor BMIs while providing visual feedback to the subject, or to control the stimulation pattern applied to the brain in DBS systems. A critical parameter for any adaptive learning algorithm is the learning rate, which dictates how fast the encoding model parameters are updated as new neural observations are received. An analytical calibration algorithm will enable achieving a predictable level of accuracy and speed in adaptive learning to improve the transient and steady-state operation of neural systems.

Closed-loop neural systems need to learn an encoding model that relates the neural signal (e.g., spikes) to the underlying brain state (e.g., motor intent) for each subject. The encoding model is often taken as a parametric function and is used to derive mathematical algorithms, termed decoders, that estimate the subject’s brain state from their neural activity. These closed-loop neural systems run in real time and often require the encoding model parameters to be learned in closed loop, online and adaptively (Fig 1). For example, in motor BMIs, neural encoding can differ for movement of the BMI compared to that of the native arm or to imagined movements [17–20]. Hence encoding model parameters are better learned adaptively in closed-loop BMI operation [17, 21–30]. Another reason for real-time adaptive learning could be the non-stationary nature of neural activity patterns over time, for example due to learning in motor BMIs [17–19], due to new experience in the hippocampus [31, 32], or due to stimulation-induced plasticity in DBS systems [14, 33, 34]. Adaptive learning algorithms in closed-loop neural systems, such as adaptive Kalman filters (KF), are typically batch-based. They collect batches of neural activity, fit a new set of parameters in each batch using maximum-likelihood techniques, and update the model parameters [22, 23, 27]. In addition to these methods, adaptive point process filters (PPF) have also been developed for tracking plasticity in offline datasets [31, 32, 35, 36]. Recently, control-based state-space algorithms have been designed for adaptive learning of point process spike models during closed-loop BMI operation, and have improved the speed of real-time parameter convergence compared with batch-based methods [28, 29].

A critical design parameter in any adaptive algorithm is the learning rate, which dictates how fast model parameters are updated based on a new observation of neural activity (Fig 1). The learning rate introduces a trade-off between the convergence time and the steady-state error of the estimated model parameters [37]. Increasing the learning rate decreases the convergence time, allowing for parameter estimates to reach their final values faster. However, this faster convergence comes at the price of a larger steady-state parameter estimation error. Similarly, a smaller learning rate will decrease the steady-state error, but lower the speed of convergence. Hence principled calibration of the learning rate is critical for fast and accurate learning of the encoding model, and consequently for both the transient and the steady-state performance of the decoder.

To date, however, adaptive algorithms have chosen the learning rate empirically. For example, in batch-based methods, once a new batch estimate is obtained, the parameter estimates from previous batches are either replaced with these new estimates [22] or are smoothly changed by weighted-averaging based on a desired half-life [23, 27]. In adaptive state-space algorithms, such as adaptive PPF, learning rate is dictated by the choice of the noise covariance in the prior model of the parameter decoder, which is again chosen empirically [28, 36, 38]. Given the significant impact of the learning rate on both the transient and the steady-state performance of closed-loop neurotechnologies, it is important to develop a principled learning rate calibration algorithm that can meet a desired error or convergence time performance for any neural recording modality (such as spikes, ECoG, and LFP) and across applications. In addition to neurotechnologies, designing such a calibration algorithm is also of great importance in general signal processing applications. Prior adaptive signal processing methods have largely focused on non-Bayesian gradient decent algorithms. These algorithms, however, do not predict the effect of the learning rate on error or convergence time (except for a limited case of scalar linear models; see Discussions) and hence can only provide heuristics for tuning the learning rate [39, 40]. A calibration algorithm that can write an explicit function for the effect of the learning rate on error and/or convergence time for both linear and nonlinear observation models would also provide a novel approach for learning rate selection in other signal processing domains [41–47]. For example, in image processing [43], in electrocardiography [41], in anesthesia control [44], in automated heart beat detection [46, 47], and in unscented Kalman filters [42], adaptive filters with learning rates are used in decoding system states or in learning system parameters in real time (see Discussions).

Here, we develop a mathematical framework to optimally calibrate the learning rate for Bayesian adaptive learning of neural encoding models. We derive the calibration algorithm both for learning a nonlinear point process model for discrete-valued spiking activity—which we term point process encoding model—, and for learning a linear model with Gaussian noise for continuous-valued neural activities (e.g., LFP or ECoG)—which we term Gaussian encoding model. Our framework derives an explicit analytical function for the effect of learning rate on parameter estimation error and/or convergence time. Minimizing the convergence time and the steady-state error covariance are competing requirements. We thus formulate the calibration problem through the fundamental trade-off that the learning rate introduces between the convergence time and the steady-state error, and derive the optimal calibration algorithm for two alternative objectives: satisfying a user-specified upper-bound on the steady-state parameter error covariance while minimizing the convergence time, and vice versa. For both objectives, we derive analytical solutions for the learning rate. The calibration algorithm can pre-compute the learning rate prior to start of real-time adaptation.

We show that the calibration algorithm can analytically solve for the optimal learning rate for both point process and Gaussian encoding models. We use extensive Monte-Carlo simulations of adaptive Bayesian filters operating on both discrete-valued spikes and continuous-valued neural observations to validate the analytical predictions of the calibration algorithm. With these simulations, we demonstrate that the learning rate selected analytically by the calibration algorithm minimizes the convergence time while satisfying an upper-bound on the steady-state error covariance or vice versa. Thus the algorithm results in fast and accurate learning of the encoding model. In addition to the encoding model, we also examine the influence of the calibration algorithm on decoding by taking a motor BMI system, which uses discrete-valued spikes or continuous-valued neural activity (e.g., ECoG or LFP), as an example. We perform extensive closed-loop BMI simulations [38, 48] that closely conform to our non-human primate BMI experiments [28, 29, 49–51] (see Discussions). Using these simulations, we show that analytically selecting the optimal learning rate can improve both the transient operation of the BMI by allowing its decoding performance to converge faster, and the steady-state performance of the BMI by allowing it to learn a more accurate decoder. We also demonstrate that large learning rates lead to inaccurate encoding models and decoders, and small learning rates delay the convergence of encoding models and decoder performance. By providing a novel analytical approach for learning rate optimization, this calibration algorithm has significant implications for closed-loop neurotechnologies and for other signal processing applications (see Discussions).

## Methods

We derive the calibration algorithm for adaptation of two widely-used neural encoding models—the linear model with Gaussian noise for continuous-valued signals such as LFP and ECoG, and the nonlinear point process model for the spiking activity. In the former case, the calibration algorithm adjusts the learning rate of an adaptive KF, and in the latter case it adjusts the learning rate of an adaptive PPF. We design the calibration algorithm for adaptive PPF and KF, as these filters have been validated in closed-loop non-human primate and human experiments both in our work and in other studies (e.g., [22, 23, 26–30]). However, to date, the learning rates in these filters have been selected using empirical tuning. Instead, the new calibration algorithm provides a novel analytical approach for selecting the learning rate to achieve a predictable and desired level of parameter error and convergence time in these widely-used adaptive filters.

**Fig 2:**
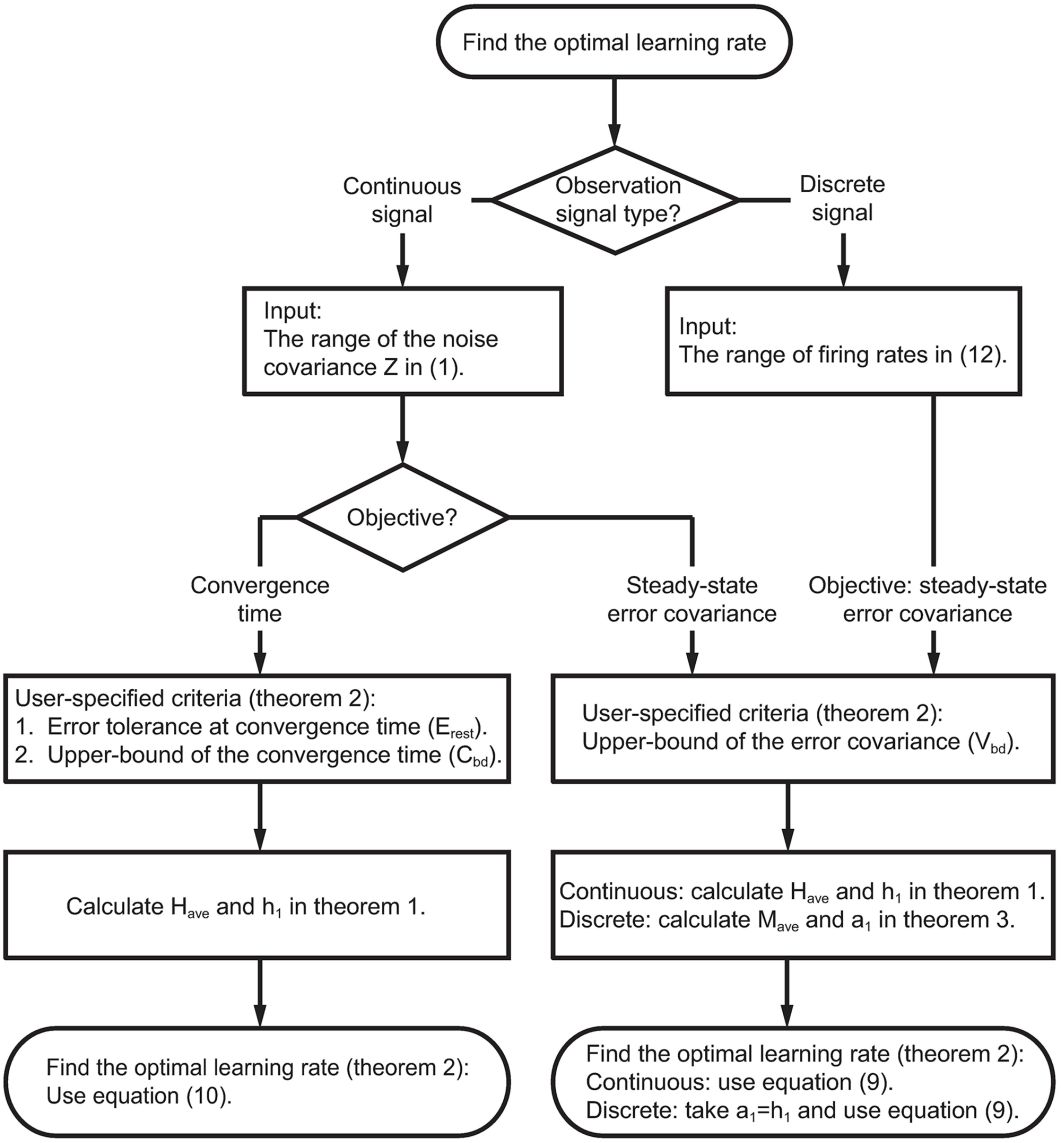
Flowchart of the calibration algorithm.

In both the adaptive PPF and the adaptive KF, the learning rate is dictated by the noise covariance of the decoder’s prior model for the parameters. In what follows, we derive calibration algorithms for two possible objectives: to keep the steady-state parameter error covariance smaller than a user-specified upper-bound while minimizing the convergence time, or to keep the convergence time faster than a user-specified upper-bound while minimizing the steady-state error covariance. We first derive analytical expressions for both the steady-state error covariance and the convergence time as a function of the learning rate by writing the recursive error dynamics and the corresponding recursive error covariance equations for the adaptive PPF and adaptive KF. By taking the limit of these recursions as time goes to infinity, we find the analytical expressions for the steady-state error covariance and the convergence time as a function of the learning rate. We then find the inverse maps of these functions, which provide the optimal learning rate for a desired objective. We also introduce the numerical simulation setup used to evaluate the effect of the calibration algorithm on both encoding models and decoding. The flowchart of the calibration algorithm is in Fig 2. Readers mainly interested in the results can skip the rest of this section.

### The calibration algorithm for continuous neural signals

In this section, we derive the calibration algorithm for continuous signals such as LFP and ECoG. We first present the observation model and the adaptive KF for these signals. We then find the steady-state error covariance and the convergence time as functions of the learning rate. Finally, we derive the inverse functions to select the optimal learning rate.

#### Adaptive KF

We denote the continuous observation signal, such as ECoG or LFP, from channel *c* by $y_{t}^{c}$. This continuous signal can, for example, be taken as the LFP or ECoG log-power in a desired frequency band as these powers have been shown to be related to the underlying brain states [52, 53]. As in various previous work (e.g., [54, 55]), we construct the continuous observation model as a linear function of the underlying brain state with Gaussian noise

$$\begin{array}{c} y_{t}^{c}={\left(\psi^{c}\right)}^{\prime }{\tilde{v}}_{t}+z_{t}^{c}. \end{array}$$ (1)

The above equation constitutes the neural encoding model for continuous neural signals where ⋅′ indicates the transpose operation, and ${\tilde{v}}_{t}={\left[1,v_{t}^{\prime }\right]}^{\prime }$ is a column vector with **v**_*t*_ denoting the encoded brain state. Also, ***ψ***^*c*^ = [*ξ*^*c*^, (***η***^*c*^)′]′ is a column vector containing the encoding model parameters to be learned. In particular, *ξ*^*c*^ is the baseline log-power and ***η***^*c*^ depends on the application. Finally, $z_{t}^{c}$ is a white Gaussian noise with variance *Z*^*c*^. As an example, in motor BMIs, we take the brain state **v**_*t*_ as the intended velocity command whether in moving one’s arm or in moving a BMI. We thus select $\eta^{c}={\left[\eta_{x}^{c},\eta_{y}^{c}\right]}^{\prime }=∥\eta^{c}∥{\left[\cos\left(\theta^{c}\right),\sin\left(\theta^{c}\right)\right]}^{\prime }$ with ‖***η***^*c*^‖ the modulation depth and *θ*^*c*^ the preferred direction of channel *c*. The goal of adaptation is to learn the encoding model parameters in (1), i.e., ***ψ***^*c*^. In some cases, it may also be desired to learn *Z*^*c*^ adaptively. Here, we first focus on adaptive learning of the parameters ***ψ***^*c*^ and the derivation of the calibration algorithm. We then present a method to learn *Z*^*c*^ concurrently with the parameters.

We write a recursive Bayesian decoder to learn the parameters ***ψ***^*c*^ recursively in real time. In neurotechnologies, such as BMIs, neural encoding model parameters are either time-invariant or change substantially slower compared with the time-scales of parameter learning (days compared with minutes, respectively; see e.g., [18, 19, 56]). Thus neural encoding model parameters in the adaptive learning algorithm can be largely assumed to be essentially fixed within relevant time-scales of parameter adaptation (e.g., minutes) in BMIs [17–29, 49–51, 57–64]. While one application of recursive Bayesian decoders (e.g., KF or PPF) is to track time-varying parameters, these filters have also been used to estimate parameters that are fixed but unknown and their application in this context has been studied extensively [65–69]. For example, the KF has been used to estimate unknown fixed parameters in prior applications such as climate modeling, control of fluid dynamics, spacecraft control, and robotics [66–69]. The PPF has also been used to estimate fixed unknown parameters [28, 29].

Assuming that all channels are conditionally independent [28, 29] (see Discussions), we can adapt the parameters for each channel separately. For convenience, we drop the superscript of the channel in what follows. A recursive Bayesian decoder consists of a prior model for the parameters, which models their uncertainty; it also consists of an observation model that relates the parameters to the neural activity. The observation model is given by (1). We build the prior model by modeling the uncertainty of ***ψ*** as a random-walk [28]

$$\begin{array}{cc} \psi_{t} & =\psi_{t-1}+s_{t}. \end{array}$$ (2)

Here **s**_*t*_ is a white Gaussian noise with covariance matrix **S** = *s***I**_*n*_(*s* > 0), where **I**_*n*_ is the identity matrix and *n* is the parameter dimension. Note that **s**_*t*_ is simply used to model our uncertainty at time *t* about the unknown parameter ***ψ*** and thus is not representing a biophysical noise. Consequently, the covariance parameter *s* is not a biophysical parameter to be learned; rather, *s* is a design choice that controls how fast parameter estimates are updated and thus serves as a tool to control the convergence time and error covariance in learning the neural encoding model parameters ***ψ*** adaptively in real time (see Appendix A in S1 Text for details). We define *s* as the learning rate since it dictates how fast parameters are updated in the Bayesian decoder as new neural observations are made [70] (see (6) below and Appendix A in S1 Text for details). Our goal is to solve for the optimal *s* that achieves a desired trade-off between the steady-state error covariance and convergence time.

Combining (1) and (2) and since both the prior and observation models are linear and Gaussian, we can derive a recursive KF to estimate ***ψ***_*t*_ from *y*_1_, ⋯, *y*_*t*_. KF finds the minimum mean-squared error (MMSE) estimate of the parameters, which is given by the mean of the posterior density. Denoting the posterior and prediction means by ***ψ***_*t*|*t*_ and ***ψ***_*t*|*t*−1_, and their covariances by **S**_*t*|*t*_ and **S**_*t*|*t*−1_, respectively, the KF recursions are given as

$$\begin{array}{cc} \psi_{t|t-1} & =\psi_{t-1|t-1} \end{array}$$ (3)

$$\begin{array}{cc} S_{t|t-1} & =S_{t-1|t-1}+S \end{array}$$ (4)

$$\begin{array}{cc} S_{t|t}^{-1} & =S_{t|t-1}^{-1}+{\tilde{v}}_{t}{\tilde{v}}_{t}^{\prime }Z^{-1} \end{array}$$ (5)

$$\begin{array}{cc} \psi_{t|t} & =\psi_{t|t-1}+S_{t|t}{\tilde{v}}_{t}Z^{-1}\left(y_{t}-{\tilde{v}}_{t}^{\prime }\psi_{t|t-1}\right). \end{array}$$ (6)

Note that **S**_*t*|*t*_ specifies the relative weight of the neural observation *y*_*t*_ compared with the previous parameter estimate in updating the current parameter estimate and thus determines how fast ***ψ***_*t*|*t*_ is learned in (6). Since **S**_*t*|*t*_ is a function of *s*, which is the only design choice in our control, we call *s* the learning rate. As shown in Appendix A in S1 Text, as *s* increases, parameters are updated faster. Hence given the encoded brain/behavioral state ${\tilde{v}}_{t}$ in a training session, we can learn the parameters adaptively using (3)–(6). To enable parameter adaptation and learning, a training session is often used in which the encoded state is measured or inferred. In our motor BMI example, the encoded brain state is the intended velocity and can be either observed or inferred behaviorally using a supervised training session in which subjects perform instructed BMI movements (e.g., [17, 21–23, 28, 29, 55, 56]) as we describe in the Numerical Simulations section. In applications such as motor BMIs, there is typically a second decoder that takes the estimated parameters from (3)–(6) to decode the brain state, e.g., the kinematics (Fig 1; see Appendix B in S1 Text). However, this brain state decoder does not affect the parameter decoder [2, 22, 23, 28]. We discuss the simulation details later in the section.

#### Overview of the two objectives for the calibration algorithm

We define ψ* as the unknown true value of the parameters ***ψ*** to be learned. Under mild conditions given in Appendix C in S1 Text, which are satisfied in our problem setup, ***ψ***_*t*|*t*_ in (6) is an asymptotically unbiased estimator (lim_*t* → ∞_ *E*[***ψ***_*t*|*t*_] = ψ*). There are two objectives that the calibration algorithm can be designed for. First, we can minimize the convergence time—defined as the time it takes for the difference (ψ*−*E*[***ψ***_*t*|*t*_]) to converge to **0**—subject to an upper-bound constraint on the steady-state error covariance of the estimated parameters. Second, we can minimize the steady-state error covariance of the estimated parameters, i.e., the Euclidean 2-norm ‖*Cov*[***ψ***_*t*|*t*_]‖, while keeping the convergence time below a desired upper-bound. We derive the calibration algorithm for each of these objectives and provide them in Theorems 1 and 2.

#### Calibration algorithm: Analytical functions to predict the effect of learning rate on parameter error and convergence time

Regardless of the objective, to derive the calibration algorithm we first need to write the error dynamics in terms of the learning rate *s*. We denote the estimation error by **g**_*t*_ = ψ*−***ψ***_*t*|*t*_. We denote the estimation error covariance at time *t* by $S_{t|t}^{*}=E\left[g_{t}g_{t}^{\prime }\right]=\text{Cov}\left[\psi_{t|t}\right]$ since ***ψ***_*t*|*t*_ is asymptotically unbiased by Appendix C in S1 Text. We denote the limit of $S_{t|t}^{*}$ in time, which is the steady-state error covariance, by $S_{+}^{*}$. Our goal is to express the steady-state error covariance $S_{+}^{*}$ and the convergence time of *E*[**g**_*t*_] as functions of the learning rate *s*.

To find the steady-state error covariance $S_{+}^{*}$ as a function of *s*, we first derive a recursive equation to compute $S_{t|t}^{*}-S_{t|t}$ from (3)–(6) as a function of the learning rate. By solving this recursive equation and taking the limit as *t* → ∞ with some approximations, we express $S_{+}^{*}$ as a function of the learning rate *s*. Similarly, by finding a recursive equation for *E*[**g**_*t*_] as a function of *s* and solving it using an approximation, we express the convergence time of E[**g**_*t*_] as a function of the learning rate *s*. To make the derivation rigorous, we assume that the encoded behavioral state **v**_*t*_ during the training session (i.e., the experimental session in which parameters are being learned adaptively) is periodic with period *T*. This holds in many cases, for example in motor BMIs in which the training session involves making periodic center-out-and-back movements [22, 23, 28]. We will show later that even in cases where the behavioral state is not periodic, our derivations of the steady-state error covariance as a function of the learning rate allow for accurate calibration to achieve the desired objectives. The derivations are lengthy and are thus provided in Appendix D in S1 Text. Also a detailed explanation of why the periodicity assumption is used in rigorous derivations, and why the approach still extends to non-periodic cases is provided in Appendix E in S1 Text. Below we present the conclusions of the derivations in the following theorem. This theorem is the basis for the calibration algorithm in the case of adaptive KF for continuous neural signal modalities.

**Theorem 1**. *Assume that the encoded state* **v**_*t*_ *in* (1) *is periodic with period T. We define* $H_{ave}=\frac{1}{T}\sum_{t=1}^{T}{\tilde{v}}_{t}{\tilde{v}}_{t}^{\prime }Z^{-1}$ *and write its eigenvalue decomposition as* **H**_*ave*_ = **U** *diag*(*h*_1_, …, *h*_*n*_)**U**′ *with* (0 < *h*_*i*_ ≤ *h*_*i*+ 1_). *We also define*

$$\begin{array}{cc} \kappa_{m} & =\frac{\sqrt{h_{m}^{2}s^{2}+4h_{m}s}-h_{m}s}{2h_{m}}\;\;\left(m=1,...,n\right). \end{array}$$

*The steady-state error covariance*, $S_{+}^{*}$, *can be expressed as a function of the learning rate s as*

$$\begin{array}{cc} S_{+}^{*} & =U\;[\begin{array}{ccc} \frac{\kappa_{1}^{2}+s\kappa_{1}}{2\kappa_{1}+s} & & \\ & \ddots & \\ & & \frac{\kappa_{n}^{2}+s\kappa_{n}}{2\kappa_{n}+s} \end{array}]\;U^{\prime }, \end{array}$$ (7)

*where* $\frac{\kappa_{m}^{2}+s\kappa_{m}}{2\kappa_{m}+s}=\frac{s}{\sqrt{h_{m}^{2}s^{2}+4h_{m}s}}=\frac{1}{\sqrt{h_{m}^{2}+\frac{4h_{m}}{s}}}$ *is monotonically increasing with respect to s*.

*The convergence dynamics of the expected error* E[**g**_*t*_] *can be expressed as a function of the learning rate s as*

$$\begin{array}{cc} E[g_{t}] & =\left(U\;[\begin{array}{ccc} \frac{\kappa_{1}}{\kappa_{1}+s} & & \\ & \ddots & \\ & & \frac{\kappa_{n}}{\kappa_{n}+s} \end{array}]\;U^{\prime }\right)\times E\left[g_{t-1}\right], \end{array}$$ (8)

*where* $\frac{\kappa_{m}}{\kappa_{m}+s}=1-\frac{\sqrt{h_{m}^{2}s^{2}+4h_{m}s}-h_{m}s}{2}$ *is monotonically decreasing with respect to s*.

*From* (8), *the behavior of the expectation of the estimation error* E[**g**_*t*_] *across time is dominated by the largest diagonal term*, $\frac{\kappa_{1}}{\kappa_{1}+s}$, *whose inverse we define as the convergence rate*.

Since **U** is the unitary matrix of the eigenvalue decomposition of **H**_*ave*_, which is not related to *s*, **U** is independent of the learning rate *s* and the diagonal terms of $S_{+}^{*}$ are strictly increasing functions of *s*. This is intuitively sound since a higher learning rate results in a larger error covariance at steady state. Also, the inverse of convergence rate in (8) is monotonically decreasing with respect to *s*. This monotonically decreasing relationship is also intuitively sound: a faster convergence rate requires a larger learning rate. These relationships clearly show the trade-off between the steady-state error covariance $S_{+}^{*}$ and the convergence time. All these properties will be confirmed in the Results section. Finally note that computing **H**_*ave*_ does not require complete knowledge of ${\tilde{v}}_{t}$ but simply the expectation (average) of a function of ${\tilde{v}}_{t}$ (e.g., simply knowing what the supervised training trajectories look like on average rather than exactly knowing the trajectories.)

Now that we have an analytical expression for the steady-state error covariance and the convergence rate as functions of the learning rate *s* ((7) and (8), respectively), all we need to do is to find the inverse of these functions to solve for the optimal learning rate *s* from a given upper-bound on $S_{+}^{*}$ or on the convergence time.

#### Calibration algorithm: The inverse functions to compute the learning rate

We now derive the inverse functions of Eqs (7) and (8) to compute the optimal learning rate *s* for each of the two objectives in the calibration algorithm. To derive the inverse function for computing the learning rate corresponding to a given steady-state error covariance, we formulate the optimization problem as that of calculating the largest learning rate *s* that satisfies $∥S_{+}^{*}∥=\lim_{t\to \infty }∥\text{Cov}\left[\psi_{t|t}\right]∥\leq V_{bd}$, where *V*_*bd*_ is the desired upper-bound on the steady-state error covariance. We want the largest learning rate that satisfies this relationship because the convergence time is a decreasing function of the learning rate and hence will benefit from larger rates. The key step in solving this inequality is observing that the 2-norm $∥S_{+}^{*}∥=\lim_{t\to \infty }∥\text{Cov}\left[\psi_{t|t}\right]∥$ is the largest singular value of $S_{+}^{*}$, which is also the largest eigenvalue of $S_{+}^{*}$ due to its positive definite property. Since the eigenvalues of $S_{+}^{*}$ are analytic functions of the learning rate in Theorem 1, we can solve the inequality analytically. The details of this derivation are shown in Appendix F in S1 Text.

For the learning rate optimization to satisfy a given convergence time upper-bound, the goal is to calculate the smallest learning rate *s* that makes $\frac{∥E\left[g_{t}\right]∥}{∥E\left[g_{0}\right]∥}\leq E_{rest}$ within the given time *C*_*bd*_, where *C*_*bd*_ is the upper-bound of the convergence time and *E*_*rest*_ is the relative estimation error (e.g., 5%) at which point we consider the parameters to have reached steady state. We want the smallest learning rate that satisfies the convergence time constraint because the steady-state error decreases with smaller learning rates. The key in solving this inequality is noting that ‖E[**g**_*t*_]‖ converges exponentially with the inverse convergence rate defined in Theorem 1. So $\frac{∥E\left[g_{t}\right]∥}{∥E\left[g_{0}\right]∥}$ can be written as a function of the learning rate *s* explicitly. The derivation details are in Appendix F in S1 Text.

We provide the conclusions of the above derivations resulting in the inverse functions for both objectives in the following theorem:

**Theorem 2** *Calibration objective 1 to constrain steady-state error: Assume that the time-step (i.e., sampling time) in* (8) *is* Δ *seconds and h*_1_ *is the smallest eigenvalue of* **H**_*ave*_ *defined in Theorem 1. The optimal learning rate to achieve an upper-bound V_bd_ on the steady-state error covariance while allowing for the fastest convergence time is given by*

$$\begin{array}{c} s=\frac{4h_{1}}{\frac{1}{V_{bd}^{2}}-h_{1}^{2}}\;\;\;with\;\;\;\frac{1}{V_{bd}^{2}}>h_{1}^{2}. \end{array}$$ (9)

*Calibration objective 2 to constrain convergence time: Define* $C_{time}=\frac{1}{4h_{1}}\times {\left(E_{rest}\right)}^{\frac{\Delta }{C_{bd}}}$, *which is independent of the learning rate s. The optimal learning rate to achieve an upper-bound C_bd_ on the convergence time, defined to be the time-point at which the relative parameter error is E_rest_, is given by*

$$\begin{array}{c} s=\frac{C_{time}}{4h_{1}^{2}}\times {\left(\frac{1}{C_{time}}-4h_{1}\right)}^{2}. \end{array}$$ (10)

To summarize, if the objective is to bound the steady-state error covariance, then the user will select the upper-bound *V*_*bd*_, calculate **H**_*ave*_ defined in Theorem 1, and apply (9) to find the optimal learning rate *s*. If the objective is to bound the convergence time, the user will select the upper-bound *C*_*bd*_, what percentage of error at convergence time they are willing to tolerate *E*_*rest*_, calculate **H**_*ave*_, and use (10) to find the optimal learning rate *s*.

#### Concurrent estimation of the noise variance *Z*

So far we have assumed that the observation noise variance, *Z*, in (1) is known (for example through offline learning). However, this variance may need to be estimated online just like the encoding parameters ***ψ***. We can address this scenario by using our knowledge of the range of possible *Z*’s, i.e., (*Z*_*min*_ and *Z*_*max*_) and use the calibration algorithm to compute the learning rate for both *Z*_*min*_ and *Z*_*max*_. Then for the first calibration objective, we can select the smaller of the two *s*’s corresponding to *Z*_*min*_ and *Z*_*max*_. This smaller *s* gives the most conservative choice to assure a given upper-bound for the steady-state error covariance. Similarly, for the second calibration objective, we can select the larger of the two *s*’s to assure a given upper-bound on the convergence time. This method is valid since the learning rate is a monotonic function of *Z*. We can see this by noting that **H**_*ave*_ in Theorem 1 is monotonic with respect to *Z*, and so are its eigenvalues (*h*_1_, …, *h*_*n*_). From (9) and (10), the learning rate *s* is also a monotonic function of *h*_1_. Together, these imply that the learning rate is a monotonic function of *Z*.

Finally, to adaptively estimate *Z* in real time, we can use the covariance matching technique [71]. Denoting $q_{t}=y_{t}-{\tilde{v}}_{t}^{\prime }\psi_{t|t-1}$, we can estimate *Z* adaptively by adding the following equation to the recursions in (3)–(6):

$$\begin{array}{cc} Z_{t|t} & =\frac{1}{L-1}\sum_{j=t-L+1}^{t}{\left(q_{j}-\bar{q}\right)}^{2}-\frac{1}{L}\sum_{j=t-L+1}^{t}{\tilde{v}}_{j}^{\prime }S_{j|j-1}{\tilde{v}}_{j}, \end{array}$$ (11)

where $\bar{q}=\frac{1}{L}\sum_{j=t-L+1}^{t}q_{j}$ is the sample mean, and *L* is the number of samples used in estimating *Z*. Here (11) is derived using the covariance matching technique. The derivation detail can be found in [71]. Since (11) only uses the prediction mean ***ψ***_*t*|*t*−1_ and the prediction covariance **S**_*t*|*t*−1_, we use (11) right after the prediction step of the KF. This means that we run the KF by first calculating the predictions using (3) and (4), then estimating *Z*_*t*|*t*_ using (11), and finally substituting *Z*_*t*|*t*_ for *Z* in (5) and (6) to get the updated parameters ***ψ***_*t*|*t*_.

### The calibration algorithm for discrete-valued spikes

We now follow the same formulation used for continuous-valued signals, such as LFP or ECoG, to derive the calibration algorithm for the discrete-valued spiking activity. The derivation follows similar steps but, due to the nonlinearity in the observation model, has some differences that we point out. Given the nonlinearities, in this case, the calibration algorithm can be derived for the main first objective, i.e., to keep the steady-state error covariance below a desired upper-bound while minimizing convergence time (Fig 2; see Discussions).

#### Adaptive PPF

The spiking activity can be modeled as a time-series of 0’s and 1’s, representing the lack or presence of spikes in consecutive time-steps, respectively. This discrete-time binary time-series can be modeled as a point process [31, 32, 48, 72–76]. A point process is specified by its instantaneous rate function. Prior work have used generalized linear models (GLM) to model the firing rate as a log-linear function of the encoded state **v**_*t*_ [36, 49, 51, 72, 74, 75], e.g., the intended velocity in a motor BMI [28, 29]. Denoting the binary spike event of neuron *c* at time *t* by $N_{t}^{c}$, and the time-step by Δ as before, the point process likelihood function is given by [72, 75]

$$\begin{array}{c} p\left(N_{t}^{c}|v_{t}\right)={\left(\lambda^{c}\left(v_{t}\right)\Delta \right)}^{N_{t}^{c}}e^{-\lambda^{c}\left(v_{t}\right)\Delta }. \end{array}$$ (12)

The above equation constitutes the neural encoding model for discrete spiking activity; here λ^*c*^(⋅) is the firing rate of neuron *c* and is taken as

$$\begin{array}{cc} \lambda^{c}\left(v_{t}\right) & =\exp(\beta^{c}+{\left(\alpha^{c}\right)}^{\prime }v_{t}), \end{array}$$ (13)

where *ϕ*^*c*^ = [*β*^*c*^, (*α*^*c*^)′]′ are the encoding model parameters to be learned. Note that the normalization constant in (12) is approximately 1 because the time-bin Δ in the discrete-time point process for spikes is taken to be small enough to at most contain one spike as shown in [75]. Thus for a small Δ, the probability of having 2 or more spikes, i.e., $p(N_{t}^{c}\geq 2)$, is negligibly small and can be ignored. So $N_{t}^{c}$ can only be either 0 or 1 and the normalization constant for 0 or 1 spikes is exactly 1. The details of this approximation can be found in [75].

For spikes, a PPF can estimate the parameters using data in a training session in which the encoded state can be either observed or inferred [28, 36, 72, 75, 77]. For example, adaptive PPF has been used to track neural plasticity in the rat hippocampus [31, 32, 77]. For motor BMIs, a closed-loop adaptive PPF has been developed to learn *ϕ*^*c*^ using an optimal feedback-control model to infer the intended velocity, resulting in fast and robust parameter convergence [28, 29]. As in the adaptive KF case, the adaptive PPF assumes that all neurons are conditionally independent so every *ϕ*^*c*^ can be updated separately [28, 36, 77] (see Discussions). From now on, we remove the superscript *c* for convenience. Denote the true unknown value of *ϕ* by ϕ*. We model our uncertainty about *ϕ* in time as a random-walk [28]

$$\begin{array}{cc} \phi_{t} & =\phi_{t-1}+q_{t}, \end{array}$$ (14)

where **q**_*t*_ is a white Gaussian noise with covariance matrix **Q** = *r***I**_*n*_(*r* > 0) and *r* is the learning rate here. Note that similar to the case of KF, **q**_*t*_ is simply used to model our uncertainty at time *t* about the unknown parameter *ϕ* and thus is not representing a biophysical noise. Consequently, the covariance parameter *r* is not a biophysical parameter to be learned but is a design choice that controls how fast neural encoding model parameters *ϕ* are learned. Thus *r* serves as the learning rate as shown in detail in Appendix A in S1 Text. Similar to the KF, the PPF has already been shown to be successful in estimating unknown fixed parameters in neurotechnologies [28, 29].

Given the observation model in (12) and the prior model in (14), adaptive PPF is derived using the Laplace approximation, which assumes that the posterior density is Gaussian. Denoting the posterior and prediction means by *ϕ*_*t*|*t*_ and *ϕ*_*t*|*t*−1_, and their covariances by **Q**_*t*|*t*_ and **Q**_*t*|*t*−1_, respectively, the adaptive PPF—derived using the Laplace Gaussian approximation to the posterior density—is given by the following recursions [28]

$$\begin{array}{cc} \phi_{t|t-1} & =\phi_{t-1|t-1} \end{array}$$ (15)

$$\begin{array}{cc} Q_{t|t-1} & =Q_{t-1|t-1}+Q \end{array}$$ (16)

$$\begin{array}{cc} Q_{t|t}^{-1} & =Q_{t|t-1}^{-1}+{\tilde{v}}_{t}{\tilde{v}}_{t}^{\prime }\lambda \left(t|\phi_{t|t-1}\right)\Delta \end{array}$$ (17)

$$\begin{array}{cc} \phi_{t|t} & =\phi_{t|t-1}+Q_{t|t}{\tilde{v}}_{t}\left[N_{t}-\lambda \left(t|\phi_{t|t-1}\right)\Delta \right] \end{array}$$ (18)

Similar to (6) in the KF, **Q**_*t*|*t*_ determines the relative weight of the neural observation *N*_*t*_ compared with the previous parameter estimate in updating the current parameter estimate and thus determines how fast *ϕ*_*t*|*t*_ is learned in (18). Because **Q**_*t*|*t*_ is governed by *r*, which is in our control, we refer to *r* as the learning rate for the PPF. As *r* increases, parameters are updated faster. Details are provided in Appendix A in S1 Text. Here $\lambda \left(t|\phi_{t|t-1}\right)=\exp\left({\tilde{v}}_{t}^{\prime }\phi_{t|t-1}\right)$ and as before ${\tilde{v}}_{t}={\left[1,v_{t}^{\prime }\right]}^{\prime }$, where **v**_*t*_ is the encoded behavioral/brain state (e.g., rat position in a maze or intended velocity in BMI), which is either observed or inferred. In studying the hippocampal place cell plasticity, for example, rat position can be observed. In motor BMIs, the intended velocity can be inferred using a supervised training session in which subjects perform instructed BMI movements [22, 23, 28, 29] as we present in the Numerical Simulations section. We now derive a calibration algorithm for the learning rate *r* in the adaptive PPF (15)–(18). The calibration algorithm minimizes the estimated parameter convergence time of *E*[*ϕ*_*t*|*t*_]→ϕ* under a given upper-bound constraint on the steady-state error covariance‖*Cov*[ϕ* − *ϕ*_*t*|*t*_]‖.

#### Calibration algorithm: Analytical function and inverse function

Learning rate calibration for spikes can again be posed as an optimization problem. We denote the error vector by **e**_*t*_ = ϕ* − *ϕ*_*t*|*t*_ and the error covariance by $\text{Cov}\left[e_{t}\right]=Q_{t|t}^{*}$. We can show that *ϕ*_*t*|*t*_, which is PPF’s estimate of the parameters, is asymptotically unbiased (lim_*t* → ∞_ *E*[*ϕ*_*t*|*t*_] = ϕ*) under some mild conditions (Appendix G in S1 Text). We define the steady-state error covariance as $Q_{+}^{*}=\lim_{t\to \infty }Q_{t|t}^{*}$. Thus the goal of the optimization problem is to select the optimal learning rate *r* that minimizes the convergence time of *E*[**e**_*t*_]→**0** while keeping the 2-norm of the steady-state error covariance $Q_{+}^{*}$ smaller than the user-defined upper-bound.

We derive the calibration algorithm similar to the case of continuous signals. We first find a recursive equation for $Q_{t|t}-Q_{t|t}^{*}$ using (15)–(18). We then solve this equation and take the limit *t* → ∞ with some approximations to write the steady-state error covariance $Q_{+}^{*}$ as an analytic function of the learning rate *r*. For rigorousness in derivations, for now we assume that the behavioral state in the training set, e.g., the intended velocity {**v**_*t*_}, is periodic with period *T*. As we also mentioned in the case of continuous signals, this assumption is reasonable in many applications such as motor BMI. However, we will show in the Results section that the calibration algorithm still works even when this assumption is violated. Also in Appendix E in S1 Text we show why the approach also extends to non-periodic cases. The derivation detail is presented in Appendix H in S1 Text. The derivation shows that the steady-state error covariance $Q_{+}^{*}$ can be written as a function of the learning rate *r* as follows:

**Theorem 3**. *Assume the encoded state* **v**_*t*_ *in* (12) *is periodic with period T. We write the eigenvalue decomposition of* $M_{ave}=\frac{1}{T}\sum_{t=1}^{T}{\tilde{v}}_{t}{\tilde{v}}_{t}^{\prime }\lambda \left(t|\phi^{*}\right)\Delta$ *as* **U** *diag*(*a*_1_, …, *a*_*n*_)**U**′ *with* (0 < *a*_*i*_ ≤ *a*_*i*+ 1_) *and we denote*

$$\begin{array}{cc} b_{m} & =\frac{\sqrt{a_{m}^{2}r^{2}+4a_{m}r}-a_{m}r}{2a_{m}}\;\;\left(m=1,...,n\right). \end{array}$$

*The steady-state error covariance*, $Q_{+}^{*}$, *can be expressed as a function of the learning rate r as*

$$\begin{array}{cc} Q_{+}^{*} & =U\;[\begin{array}{ccc} \frac{b_{1}^{2}+b_{1}r}{2b_{1}+r} & & \\ & \ddots & \\ & & \frac{b_{n}^{2}+b_{n}r}{2b_{n}+r} \end{array}]\;U^{\prime }. \end{array}$$ (19)

Compared with the steady-state error covariance $S_{+}^{*}$ for continuous signals in (7), the steady-state error covariance for spikes $Q_{+}^{*}$ in (19) has exactly the same form when replacing *h*_*i*_ with *a*_*i*_ and *s* with *r*. Hence to compute the optimal learning rate *r* from (19), we can again apply (9) while replacing *h*_*i*_ with *a*_*i*_ and *s* with *r*. Note that **M**_*ave*_ includes the firing rate λ(*t*|ϕ*), which is related to the unknown true parameter ϕ*. Since λ(*t*|ϕ*)Δ in **M**_*ave*_ has the same role as *Z*^−1^ in **H**_*ave*_ for KF, and since (19) has the same form as (7), the learning rate *r* is a monotonic function of λ(*t*|ϕ*)Δ similar to the case of *Z* for KF. Thus we use our knowledge of the minimum and maximum possible firing rates to calculate the extreme values of the learning rate *r* from (9), and select the minimum of the two *r*’s as the most conservative value to keep the steady-state error covariance under the given bound *V*_*bd*_.

### Calibration algorithm for non-periodic state evolution

For both discrete and continuous signals, we considered a periodic behavioral state (e.g., intended velocity) in the training data for the derivations to satisfy the mild conditions in Appendix C in S1 Text. However, the derivation of (7), (8) and (19) are based on **H**_*ave*_ and **M**_*ave*_ for the continuous and discrete signals, respectively, which are simply the average values of functions of the state {**v**_*t*_}. So the core information needed in the calibration algorithm is not the state periodicity, but its expected value, which we can compute empirically for any state evolution. As detailed in Appendix E in S1 Text, the periodicity of **v**_*t*_ is simply required to ensure that the mean of the prediction covariance **S**_*t*+ 1|*t*_ is well-defined at steady state. If we ignore some mathematical rigorousness and instead assume that **S**_*t*+ 1|*t*_ has bounded steady-state moments (which is a relatively mild requirement), then this calibration algorithm can be generalized to the case with non-periodic **v**_*t*_ directly. That is precisely why, as we show using simulations in the Results section, the calibration algorithm works even in the case of random evolution for the states {**v**_*t*_} in the training experiment. Periodicity is simply required to guarantee the *existence* of the mean of **S**_*t*+ 1|*t*_ at steady state (instead of assuming this existence) in the derivations, as detailed in Appendix E in S1 Text.

### Numerical simulations

To validate the calibration algorithm, we run extensive closed-loop numerical simulations. We show that the calibration algorithm allows for fast and precise learning of encoding model parameters, and subsequently for a desired transient and steady-state behavior of the decoders (Fig 1). While the calibration algorithm can be applied to learn encoding models and decoders for any brain state, as a concrete example, we use a motor BMI to validate the algorithm.

In motor BMIs, the relevant brain state is the intended movement. The BMI needs to learn an encoding model that relates the neural activity to the subject’s intended movement. We simulate a closed-loop BMI within a center-out-and-back reaching task with 8 targets. In this task, the subject needs to take a cursor on a computer screen to one of 8 peripheral targets, and then return it to the center to initiate another trial [29, 56]. To simulate how subjects generate a pattern of neural activity to control the cursor, we use an optimal feedback-control (OFC) model of the BMI that has been devised and validated in prior experiments [28, 29, 48, 49] and is inspired by the OFC models of the natural sensorimotor system [78–80]. We then simulate the spiking activity as a point process using the nonlinear encoding model in (12) and simulate the ECoG/LFP log-powers as a Gaussian process linearly dependent on the brain state [55] using the linear encoding model in (1). We test the calibration algorithm for adaptive learning of the ECoG/LFP and the spike model parameters. We assess the ability of the calibration algorithm to enable fast and accurate learning of the encoding models, and to lead to a desired transient and steady-state performance of the decoder.

To simulate the intended movement, we use the OFC model. We assume that movement evolves according to a linear dynamical model [28, 29, 48, 49]

$$\begin{array}{c} x_{t+1}=Ax_{t}+Bu_{t}+w_{t}, \end{array}$$ (20)

where $x_{t}={\left[d_{t}^{\prime },v_{t}^{\prime }\right]}^{\prime }$ is the kinematic state at time *t*, with **d**_*t*_ and **v**_*t*_ being the position and velocity vectors in the two-dimensional space, respectively. Here **u**_*t*_ is the control signal that the brain decides on to move the cursor and **w**_*t*_ is white Gaussian noise with covariance matrix **W**. Also, **A** and **B** are coefficient matrices that are often fitted to subjects’ manual movements [22, 23, 28, 29, 56, 80]. Similar to prior work [28, 29, 48, 49], we write (20) as

$$\begin{array}{c} {}[\begin{array}{c} d_{1}\left(t+1\right)\\ d_{2}\left(t+1\right)\\ v_{1}\left(t+1\right)\\ v_{2}\left(t+1\right) \end{array}]=[\begin{array}{cccc} 1 & 0 & \Delta & 0\\ 0 & 1 & 0 & \Delta \\ 0 & 0 & \alpha & 0\\ 0 & 0 & 0 & \alpha \end{array}][\begin{array}{c} d_{1}\left(t\right)\\ d_{2}\left(t\right)\\ v_{1}\left(t\right)\\ v_{2}\left(t\right) \end{array}]+[\begin{array}{cc} 0 & 0\\ 0 & 0\\ 1 & 0\\ 0 & 1 \end{array}][\begin{array}{c} u_{1}\left(t\right)\\ u_{2}\left(t\right) \end{array}]+[\begin{array}{c} 0\\ 0\\ w_{1}\left(t\right)\\ w_{2}\left(t\right) \end{array}], \end{array}$$ (21)

where Δ is the time-step and *α* is selected according to our prior non-human primate data [28, 29].

The OFC model assumes that the brain quantifies the task goal within a cost function and decides on its control commands by minimizing this cost. For the center-out movement task, the cost function can be quantified as [28, 29, 48, 49, 78, 80]

$$\begin{array}{c} J=\sum_{t=1}^{\infty }{∥d_{t}-d^{*}∥}^{2}+w_{v}{∥v_{t}∥}^{2}+w_{r}{∥u_{t}∥}^{2}, \end{array}$$ (22)

where d* is the target position, and *w*_*v*_ and *w*_*r*_ are weights selected to fit the profile of manual movements. For the linear dynamics in (20) and the quadratic cost in (22), the optimal control command is given by the well-known infinite horizon linear quadratic Gaussian (LQG) solution as [28, 29, 48, 49, 81]

$$\begin{array}{c} u_{t}=-L\left(x_{t}-x^{*}\right), \end{array}$$ (23)

where x* = [d*′, **0**′]′ is the target for position and velocity (as the subject needs to reach the target position and stop there). Here **L** is the gain matrix, which can be found recursively and offline by solving the discrete-time Riccati equation [81]. By substituting (23) in (20), we can compute the intended kinematics of the subject in response to visual feedback of the current decoded cursor kinematics **x**_*t*_ in our simulations [28]. Details are provided in our prior work [28, 38, 48]. Note that we use a single OFC model to simulate the brain strategy throughout all closed-loop numerical simulations—i.e., both during training experiments in which parameters are being learned in parallel to the kinematics being decoded (Fig 1), or after training is complete and during pure decoding experiments when the learned parameters are fixed and the learned decoder is used to move the cursor. Indeed prior work have suggested that the brain strategy in closed-loop control largely remains consistent, e.g., regardless of whether parameters are being adapted or not (e.g., [22, 23, 26, 28, 29, 49, 82, 83]).

The subject’s intended velocity **v**_*t*_ is in turn encoded in neural activity. We first test the performance of the calibration algorithm for continuous ECoG/LFP recordings. We then test this performance for discrete spike recordings.

For the continuous signals, we simulate 30 LFP/ECoG features whose baseline powers and preferred directions in (1) are randomly selected in [1, 6] dB and [0, 2*π*], respectively. The modulation depth, ‖***η***‖, in each channel is randomly-selected in [7, 10] and the noise variances are randomly-selected in [320, 380]. The initial value, ***ψ***_0|0_, and the true value, ψ*, of each channel are selected randomly and independently. The eight targets are around a circle with radius 0.3. Each trial including the forward and the back movement for a selected target in the center-out-and-back task takes 2 secs. During the training experiment, the subject reaches the targets in the counter-clockwise order repeatedly. To assess whether the calibration algorithm can analytically compute the steady-state error covariance and convergence time for a given learning rate accurately, we simulate 3000 trials under each learning rate considered.

For spikes, we simulate 30 neurons. Here since the state **v**_*t*_ is the intended velocity, we can also interpret (13) as a modified cosine-tuning model [75, 84] by writing it as

$$\begin{array}{c} \lambda^{c}\left(v_{t}\right)=\exp\left(\beta^{c}+∥\alpha^{c}∥∥v_{t}∥\cos\left(\theta_{t}-\theta^{c}\right)\right), \end{array}$$ (24)

where *θ*_*t*_ is the direction of **v**_*t*_, *θ*^*c*^ is the preferred direction of the neuron (or direction of ***α***^*c*^ = ‖***α***^*c*^‖[cos *θ*^*c*^, sin *θ*^*c*^]′), and finally ‖***α***^*c*^‖ is the modulation depth. For each neuron, we select the baseline firing rate randomly between [4, 10] Hz and the maximum firing rate randomly between [40, 80] Hz. We select each neuron’s preferred direction in (24) randomly between [0, 2*π*]. The task setup is equivalent to the continuous signal case. We simulate 1000 trials for each learning rate considered.

We also examine the effect of the calibration algorithm on kinematic decoding. For continuous signals, we use a KF kinematic decoder as in prior work (e.g., [22, 23, 55]). For the discrete spike signals, we use a PPF kinematic decoder as in prior work (e.g., in real-time BMIs [28, 29]). Kinematic decoder details have also been provided in Appendix B in S1 Text for convenience.

## Results

We first investigate whether the calibration algorithm can analytically approximate two quantities well: the steady-state error covariance and the convergence time of the encoding model parameters as a function of the learning rate. We do so by running multiple closed-loop BMI simulations with different learning rates. These Monte-Carlo simulations allow us to compute the true value of the two quantities. We then compare these true values with the analytically-computed values from the calibration algorithm. We find that, for both continuous and discrete signals, the calibration algorithm accurately computes its desired quantity (i.e, either the error covariance or the convergence time) for any type of behavioral state trajectory in the training data (i.e., periodic or not). Thus the calibration algorithm can find the optimal learning rate for a desired trade-off between the parameter convergence time and error covariance. We also show how the inverse function can be used to compute the learning rate for a desired trade-off. Moreover, we examine how the calibration algorithm—and consequently the learned encoding model—affects decoding performance. We show that, by finding the optimal learning rate, the calibration algorithm results in fast and accurate decoding. In particular, compared to the optimal rate, larger learning rates could result in inaccurate steady-state decoding performance and smaller learning rates result in slow convergence of the decoding performance.

### The calibration algorithm computes the convergence time and error covariance accurately with continuous signals

We first assess the accuracy of the analytically-computed error covariance and convergence time by the calibration algorithm. As described in detail in Numerical Simulation section, we run a closed-loop BMI simulation in which the subject performs a center-out-and-back task to eight targets in counter-clockwise order. We simulate 30 LFP/ECoG features.

**Fig 3:**
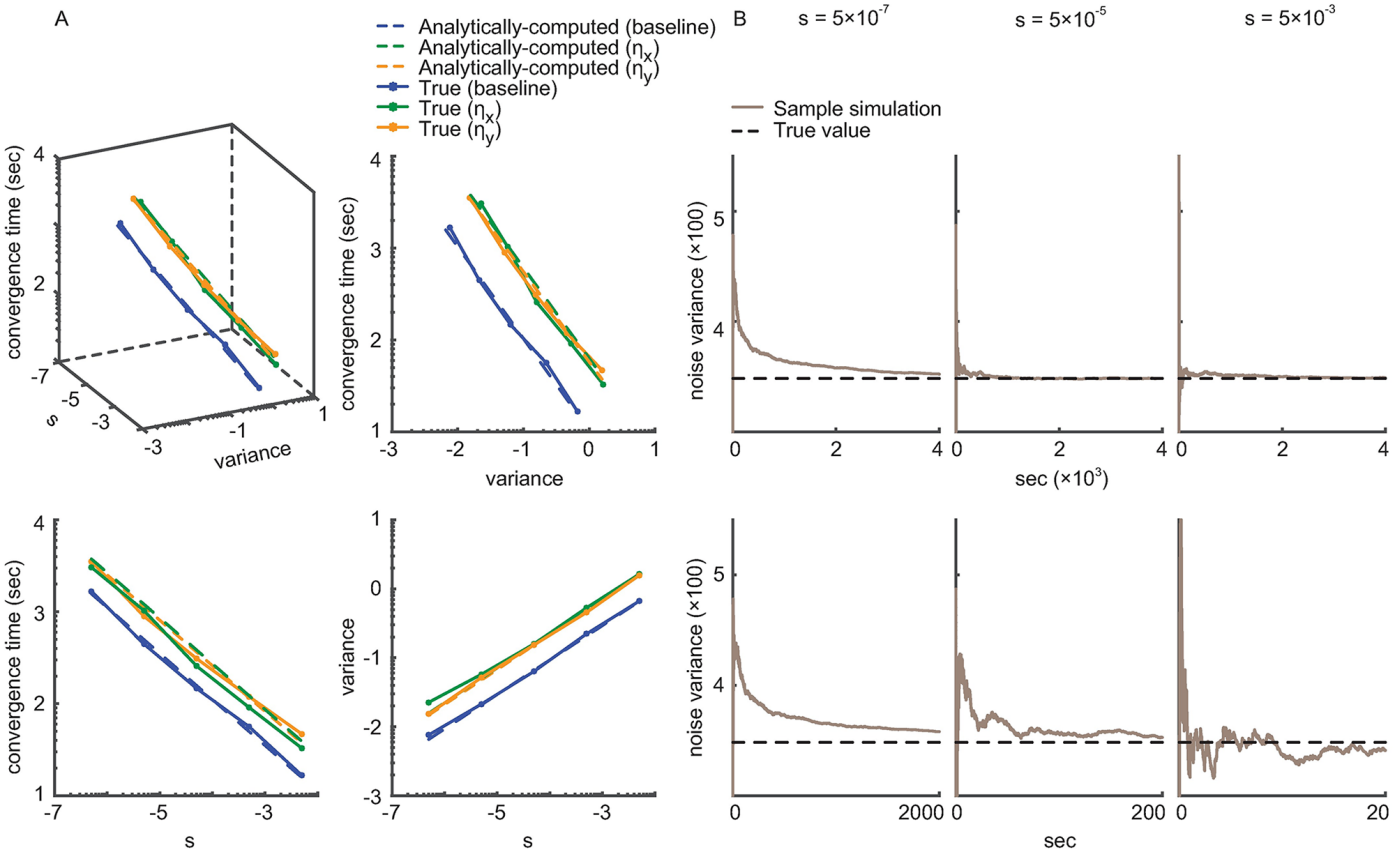
The calibration algorithm accurately computes the steady-state error covariance and convergence time as a function of learning rate for continuous signals. (A) The analytically-computed and the true error covariance and convergence time of the encoding model parameters (baseline, ***η***_*x*_, and ***η***_*y*_ in (1)) for different learning rates *s*, across a wide range of *s*. The top left panel shows the relation between the three quantities. The other three panels are projections of this plot to three planes, showing each of the three pair-wise relationships. All axes are in log scale. True quantities are computed from BMI simulations with periodic center-out-and-back training datasets. The analytically-computed values are obtained by the calibration algorithm according to Eqs (7) and (8). The analytically-computed and true values match tightly across a wide range of learning rates, showing that the calibration algorithm can accurately compute the learning rate for a desired trade-off between steady-state error and convergence time. (B) Adaptive estimation of the unknown observation noise variance using (11) under different learning rates *s*. The bottom three panels are zoomed-in versions of the top panels to show the transient behavior of the estimated noise variance, which converges to its true value in all cases.

We define the convergence time as the time when the estimated parameters reach within 5% of their true values, i.e., ‖***ψ***_*t*|*t*_ − ψ*‖≤0.05 × ‖***ψ***_0|0_ − ψ*‖ (so *E*_*rest*_ = 0.05; as defined before ***ψ***_*t*|*t*_, ψ*, and ***ψ***_0|0_ are the current parameter estimate, the true parameter value, and the initial parameter estimate, respectively.) Fig 3A shows the true and the analytically-computed error covariance and convergence time as a function of the learning rate, across a wide range of learning rates. The analytically-computed values are close to the true values. From Fig 3A, the average normalized root-mean-squared errors (RMSE) between the true and the analytically-computed values for the convergence time and the steady-state error covariance are 3.6% and 1.6%, respectively (where normalization is done by dividing by the range of possible convergence time and covariance values). Fig 3A shows that as the learning rate *s* increases, the error covariance increases and the convergence time decreases. Also, the error covariance is inversely related to the convergence time. These trends also demonstrate the fundamental trade-off between steady-state error covariance and convergence time.

In the above analysis, we considered estimating the encoding model parameters ***ψ***_*t*|*t*_ in (6). As derived in (11), when the noise variance *Z* in (1) is unknown, we can also estimate this variance in real time and simultaneously with the parameters. We thus repeated our closed-loop BMI simulations, this time simultaneously estimating the noise variance *Z*_*t*|*t*_ to show that it converges to the true value regardless of the learning rate *s*. Fig 3B shows that *Z*_*t*|*t*_ converges to the true value with all tested learning rates, which cover a large range (5 × 10^−7^ to 5 × 10^−3^). Moreover, even when estimating both ***ψ***_*t*|*t*_ and the noise variance *Z*_*t*|*t*_ jointly, the analytically-computed error covariance is still close to the true one (normalized RMSE is 4.5%). Overall, the analytically-computed error covariance is robust to the uncertainty in *Z*_*t*|*t*_ because *Z*_*t*|*t*_ converges to the true value at steady state regardless of the learning rate (Fig 3B).

### Use of the inverse function to compute the learning rate

Here we show how the inverse functions in Theorem 2 can be used to select the learning rate. In our example, we require the 95% confidence bound of the estimated encoding model parameters (i.e., ±2 standard deviations of error) to be within 10% of their average value. Thus this constraint provides the desired upper-bound on the steady-state error covariance *V*_*bd*_. In general, *V*_*bd*_ can be selected in any manner desired by the user. Once *V*_*bd*_ is specified, we use (9) and find the optimal value of the learning rate as *s*_1_ = 5.6 × 10^−5^. Hence the calibration algorithm dictates that the learning rate should be smaller than *s*_1_ to satisfy the desired error covariance upper-bound.

Let’s now suppose that we want to ensure that the convergence time is within a given range. In our example, we require the estimation error to converge within 7 minutes, where convergence is defined as reaching within 5% of the true value (*E*_*rest*_ = 0.05). This constraint sets the upper-bound on the convergence time to be *C*_*bd*_ = 7min = 420 sec. The calibration algorithm using (10) dictates that the learning rate needs to be larger than 4.75 × 10^−5^.

**Fig 4:**
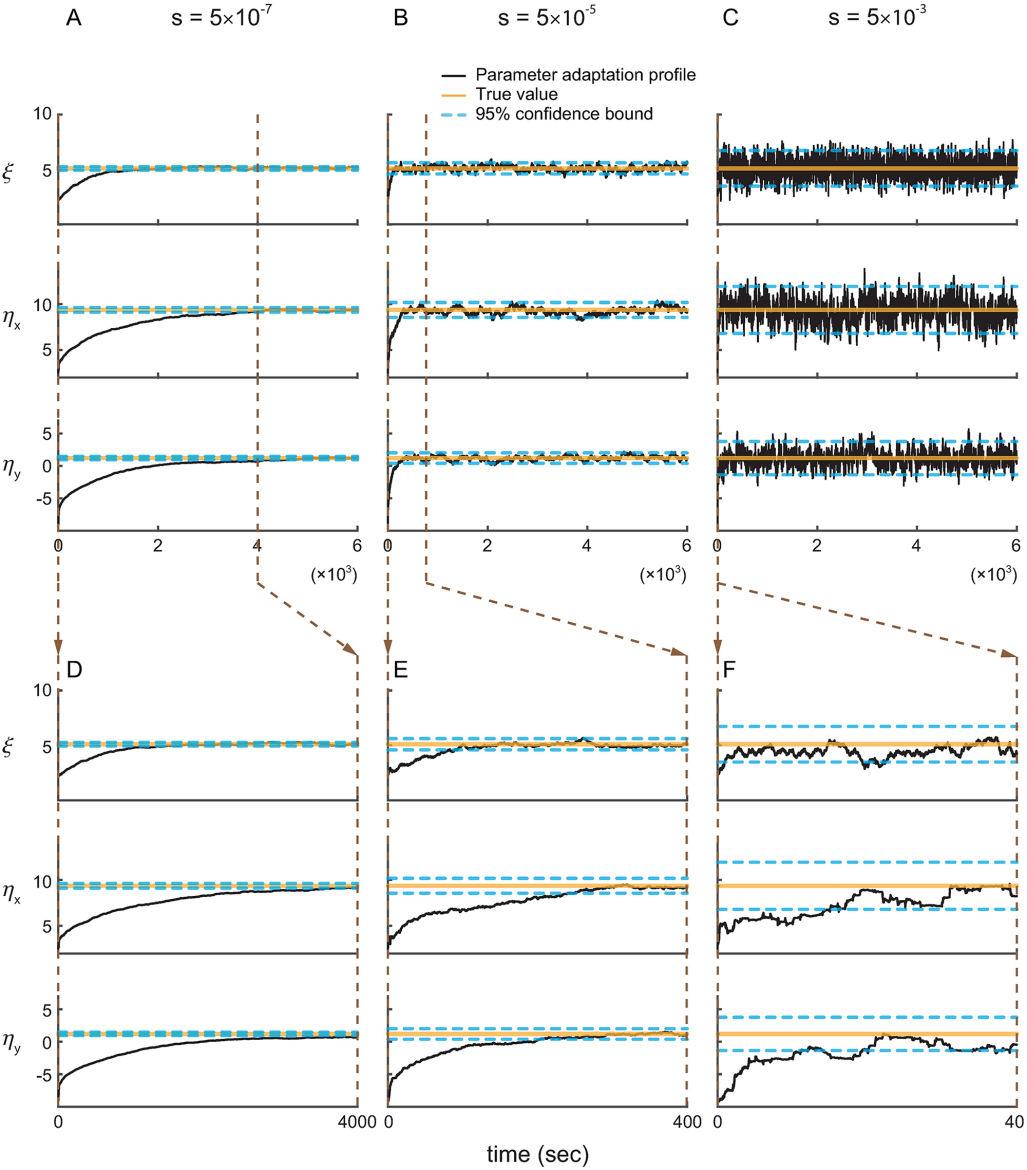
Parameter adaptation profiles confirm the accuracy of the calibration algorithm with continuous signals. (A–C) show sample adaptation profiles of the model parameters ***ψ***_*t*|*t*_ for different learning rates *s* in ascending order. For each learning rate, the estimated parameters are within the analytically-computed 95% confidence bounds by the calibration algorithm about 96% of the time, demonstrating the accuracy of the calibration algorithm.

Taken together, for the above constraints for error covariance and convergence time, any learning rate 4.75 × 10^−5^ < *s* < 5.6 × 10^−5^ is admissible. For conciseness and as an illustrative example, we select the learning rate *s* = 5 × 10^−5^, which satisfies both criteria above. In the next section, we examine the effect of this learning rate on the estimated model parameters over time, i.e., on the adaptation profiles (Fig 4).

### Parameter adaptation profiles confirm the accuracy of the calibration algorithm

We also examined the evolution of the estimated encoding model parameters ***ψ***_*t*|*t*_ in time, which we refer to as the parameter adaptation profiles. Plotting the adaptation profile provides a direct way of investigating the influence of the learning rate on the estimated encoding model. We plot the adaptation profiles for the optimal learning rate in our example above, i.e., *s* = 5 × 10^−5^. We also show these profiles for a smaller and a larger learning rate (Fig 4). We used these adaptation profiles to further assess the accuracy of the calibration algorithm.

The adaptation profiles confirm the accuracy of the calibration algorithm as expected from Fig 3A. We used (7) to find the steady-state error covariance for each learning rate in Fig 4 and consequently to compute the 95% confidence bounds for the parameter estimates (which are equal to ±2 square-root of the analytically-computed error covariance). We then empirically found the percentage of time during which the steady-state parameter estimates were within this 95% bound. If the covariance matrix is accurately computed by the calibration algorithm, then this percentage should be close to 95%. We found that about 96% of the time, the steady-state estimated parameters lie within the 95% confidence bound calculated by the calibration algorithm for all learning rates. Finally, we also simulated the case where the parameters may shift from day to day (see Discussions) to see the application of the calibration algorithm in this case. We confirmed, as shown in S1 Fig, that the same KF with a learning rate calculated from the calibration algorithm (Fig 4B) can track the parameters and satisfy the criteria on steady-state error and convergence time on both days.

### The calibration algorithm generalizes to different state evolution profiles

**Fig 5:**
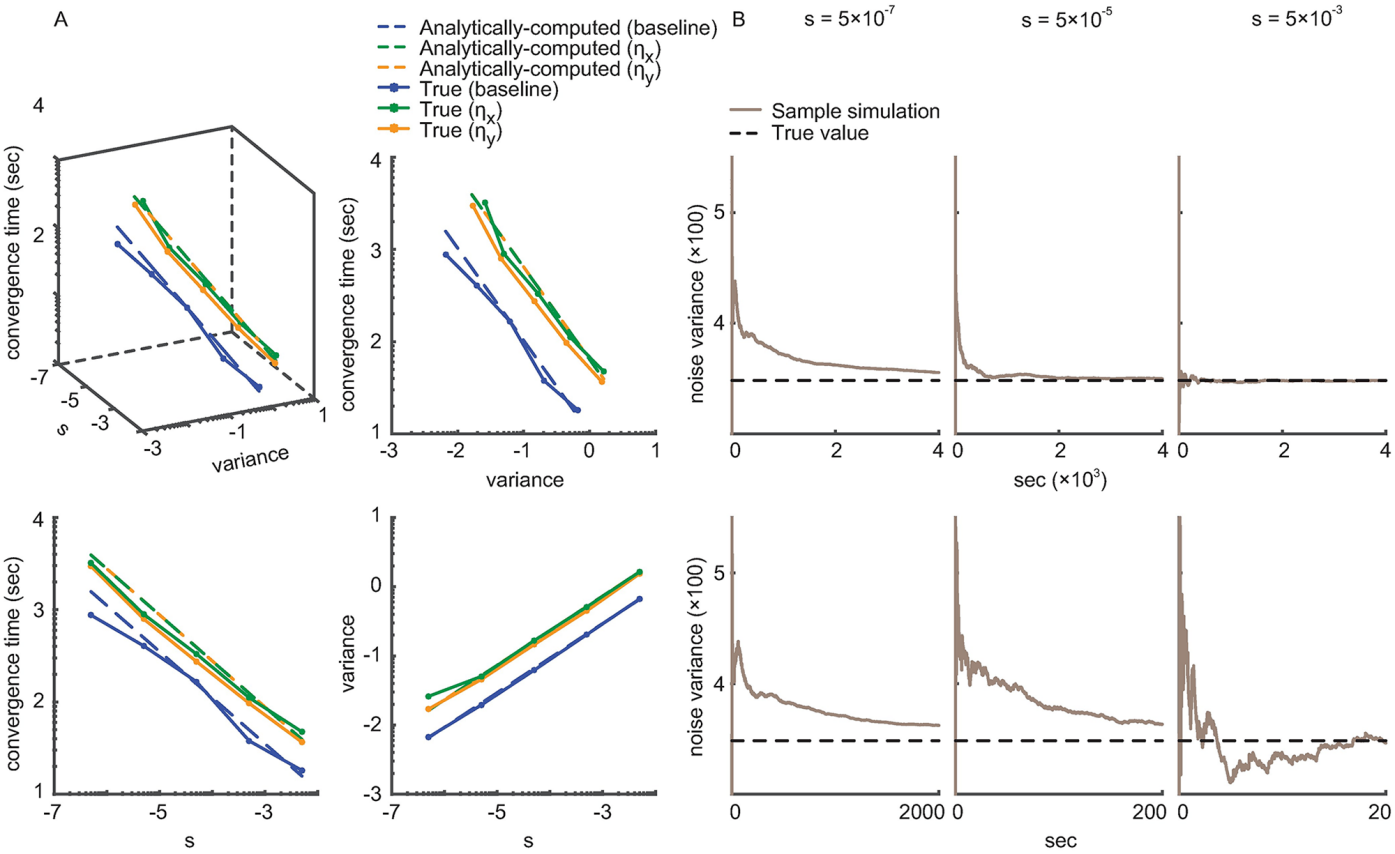
The calibration algorithm generalizes to training datasets with non-periodic state trajectories. Figure convention is the same as Fig 3. Here the true quantities are computed in closed-loop BMI simulations with a non-periodic trajectory generated by selecting targets randomly and uniformly. The analytically-computed error covariance and convergence times given by the calibration algorithm closely match their true values across a wide range of the learning rate *s*, showing that the calibration algorithm extends across training datasets with different state-evolution trajectories.

In the algorithm derivation and for rigorousness to ensure the existence of the mean of the prediction covariance **S**_*t*+ 1|*t*_ at steady state (instead of simply assuming this existence; Appendix E in S1 Text), we assume that the evolution of behavioral state {**v**_*t*_}, e.g., the trajectory, is periodic in the training data. However, in computing the error covariance and the convergence time, the only aspect of **v**_*t*_ needed by the calibration algorithm is not periodicity, but an average of a function of **v**_*t*_ over time, which is **H**_*ave*_. Indeed, if we assume **S**_*t*+ 1|*t*_ has bounded steady-state moments, then our derivation directly applies to the general non-periodic case (Appendix E in S1 Text, S2 Fig). To show that the calibration algorithm also extends to the case of non-periodic state evolutions, we run a closed-loop BMI simulation with a non-periodic trajectory. In this simulation, in each trial, one of eight targets is instructed randomly according to a uniform distribution over the targets. So the trajectory is no longer periodic (in contrast to when the targets are instructed one by one and in counter-clockwise order). The comparison between the true error covariance and convergence time and their values computed analytically by the calibration algorithm are shown in Fig 5A, across a wide range of learning rates. The analytically-computed values are still close to the true values, with an average normalized RMSE of 2.1% and 7.4% for the steady-state error covariance and the convergence time, respectively. Similarly, when the noise variance *Z* needs to be estimated, its estimate *Z*_*t*|*t*_ from (11) still converges to the true value for all learning rates (Fig 5B). Even when estimating *Z*_*t*|*t*_ simultaneously with parameters, the calibration algorithm can approximate the error covariance well (normalized RMSE is 2.6%). Taken together, these results demonstrate that the calibration algorithm can generalize to a wide range of problems since the training state-evolution when adapting the encoding models could have a general form.

### The calibration algorithm for discrete spiking activity

**Fig 6:**
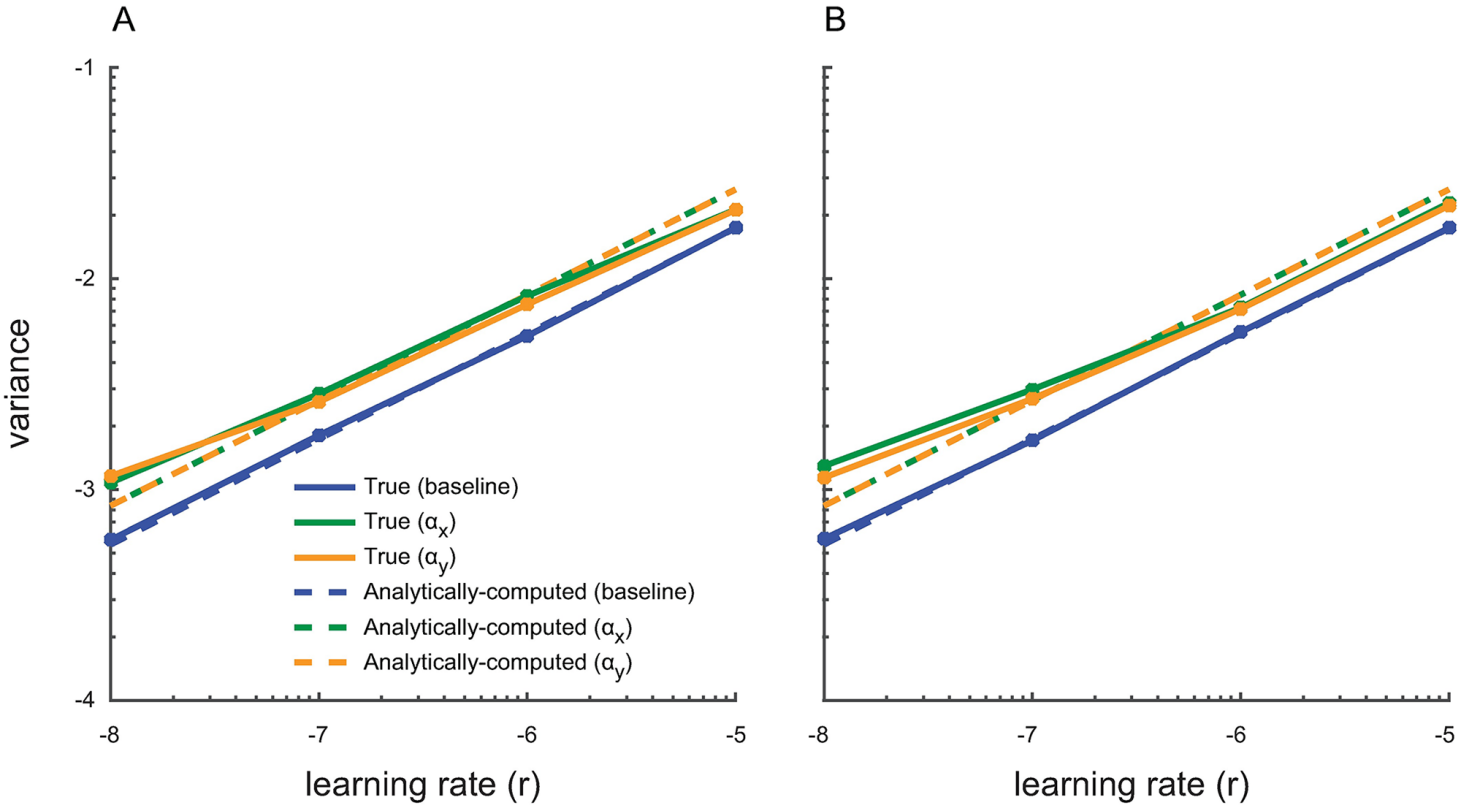
The calibration algorithm accurately computes the steady-state error covariance for discrete spiking activity. (A) The analytically-computed and the true steady-state error covariance as a function of the learning rate *r*. True values are found from closed-loop BMI simulations with a periodic center-out-and-back trajectory. The calibration algorithm analytically computes the covariance based on (19). The calibration algorithm closely approximates the steady-state error covariance as demonstrated by the closeness of the analytically-computed and true curves across a wide range of *r*. (B) Figure convention is the same as (A) except that all true values are computed in closed-loop BMI simulations with a non-periodic trajectory generated by selecting one of the eight targets randomly and uniformly in each trial. The calibration algorithm can again closely approximate the steady-state error covariance, demonstrating the generalizability of the approach to training datasets with varying state-evolution trajectories.

We also validate the calibration algorithm for discrete-valued spiking observations. We run multiple closed-loop BMI simulations with either a periodic or a non-periodic trajectory. The simulation setting is the same as that for continuous signals and given in Numerical Simulation section. Fig 6 shows that the analytically-computed error covariance is close to its true value across a wide range of learning rates with any type of trajectory (i.e., periodic or not). The average normalized RMSE between the true and the analytically-computed error covariance is around 5% with either periodic or non-periodic trajectory. This result shows that the calibration algorithm can also accurately compute the learning rate effect for a nonlinear point process model of spiking activity. The result also verifies the generality of the calibration algorithm to different state evolution profiles during adaptation, as was the case for continuous signals.

In the case of spikes, the inverse function can again be used to select the learning rate for a given upper-bound on the steady-state error covariance. For example, we can require the error covariance to be within 7% of the average values for all parameters, which provides the value of *V*_*bd*_. Again, *V*_*bd*_ can be selected as desired by the user. Once *V*_*bd*_ is specified, we use the inverse function using Theorem 3 and Eq (9) and find that the corresponding optimal learning rate *r* is 10^−7^.

**Fig 7:**
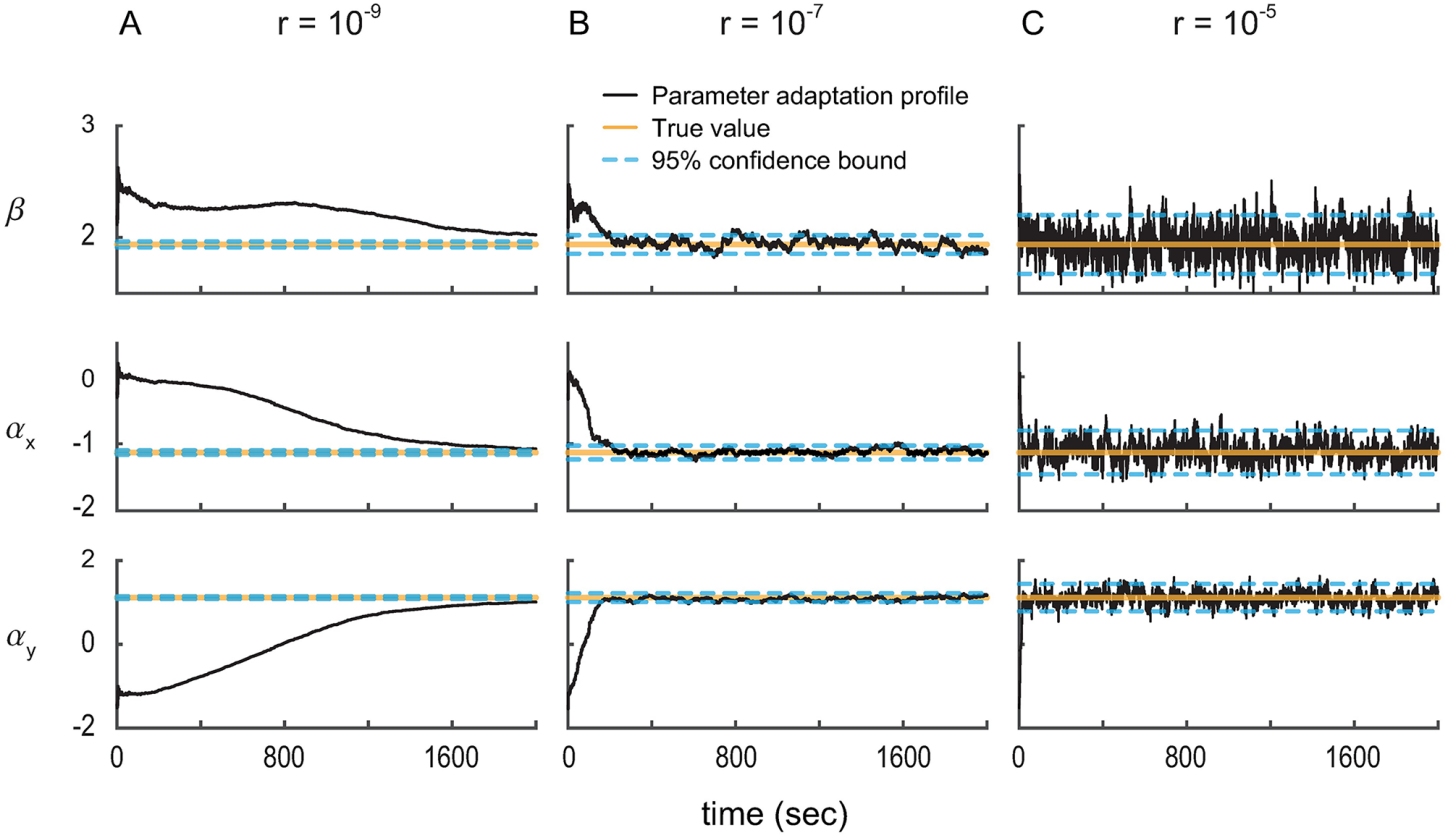
Parameter adaptation profiles confirm the accuracy of the calibration algorithm with discrete spiking activity. (A)–(C) show sample adaptation profiles of model parameters *ϕ*_*t*|*t*_ in a closed-loop BMI simulation under different learning rates *r* in ascending order. Increasing the learning rate increases the error covariance. Also, about 96% of the time, the parameter estimates at steady state are within the 95% confidence bounds computed by the calibration algorithm; this demonstrates that the calibration algorithm can closely approximate the error covariance and consequently the confidence bounds.

We also confirm the accuracy of the calibration algorithm using the parameter adaptation profiles. We plot three realizations of the estimated point process parameters, *ϕ*_*t*|*t*_, under different learning rates *r* to examine whether the 95% confidence bounds computed by the calibration algorithm are accurate (Fig 7; similar analysis to the case of continuous signals). Note that the confidence bounds are given by twice the square-root of the analytically-computed covariance matrix. We use the optimal learning rate computed for our example above, i.e., *r* = 10^−7^, and a smaller and a larger learning rate in Fig 7. We find that at steady state, the estimated parameters are within the 95% confidence bound about 96% of time. This shows the accuracy of the analytically-computed confidence bound (if this bound is correct, about 95% of the time the estimates should be within confidence bounds). This result is consistent with the good match between the true and analytically-computed covariances in Fig 6.

Finally, even though the convergence time cannot be analytically obtained in the case of spike observations, it is still significantly affected by the learning rate *r*. For a small learning rate (*r* = 10^−9^), the parameter estimate *ϕ*_*t*|*t*_ does not converge to its true value even in 2000 sec. In comparison, this convergence time is only about 200 sec for an intermediate learning rate (*r* = 10^−7^). Hence to allow for fast convergence, it is critical to select the maximum possible learning rate that satisfies a desired upper-bound constraint on error covariance. This was the basis for the calibration algorithm.

### The effect of learning rate on decoding

The selection of the optimal learning rate is critical not only for fast and accurate estimation of the encoding model, but also for accurate decoding of the brain state. Here we show that the selection of the appropriate learning rate by the calibration algorithm can improve both the transient and the steady-state operation of decoders. We simulate closed-loop BMI decoding under various learning rates. Since the optimal trajectory for reaching a target in a center-out task should be close to a straight line connecting the center to the target, as the measure of decoding accuracy we use the RMSE between the decoded trajectory and these straight lines [22, 23, 28, 29, 56] (the error is the perpendicular distance of the decoded position to the straight line at each time).

**Fig 8:**
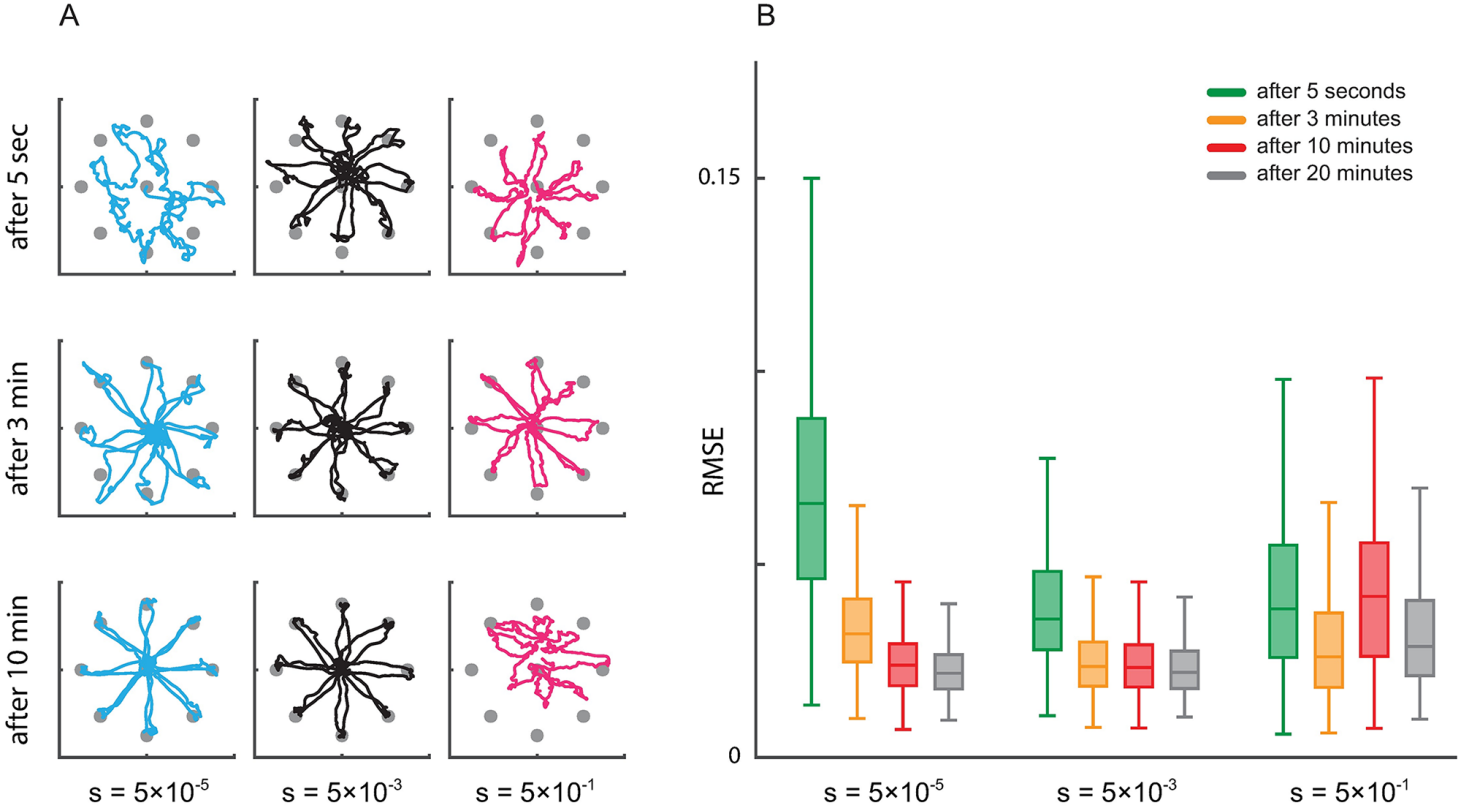
Learning rate calibration affects both the transient and the steady-state performance of closed-loop BMI decoders with continuous neural activity. (A) The evolution of the decoded trajectory as the adaptation time is increased under different learning rates *s*. Note that the decoder is fixed after a given adaptation time is completed (as noted on each row). The fixed decoder is then used to generate the displayed trajectories. Each color corresponds to one learning rate. Decoding performance is unstable when the learning rate is large (*s* = 5 × 10^−1^) even at steady state; this means that depending on exactly when we stop the adaptation and fix the decoder, performance widely oscillates due to the large steady-state model parameter error. (B) RMSE of the decoded trajectory under different learning rates for different adaptation times. RMSE is computed for a fixed decoder that was obtained by stopping the adaptation at various times (different colors). RMSE converges faster as the learning rate is increased (*s* = 5 × 10^−5^ to 5 × 10^−3^, for example). However, if the learning rate is selected too large (*s* = 5 × 10^−1^), RMSE oscillates depending on when adaptation is stopped, without converging to a stable number. These results show that appropriately calibrating the learning rate is important not only for encoding model estimation but also for a desired trade-off between convergence time and steady-state RMSE in decoding.

**Fig 9:**
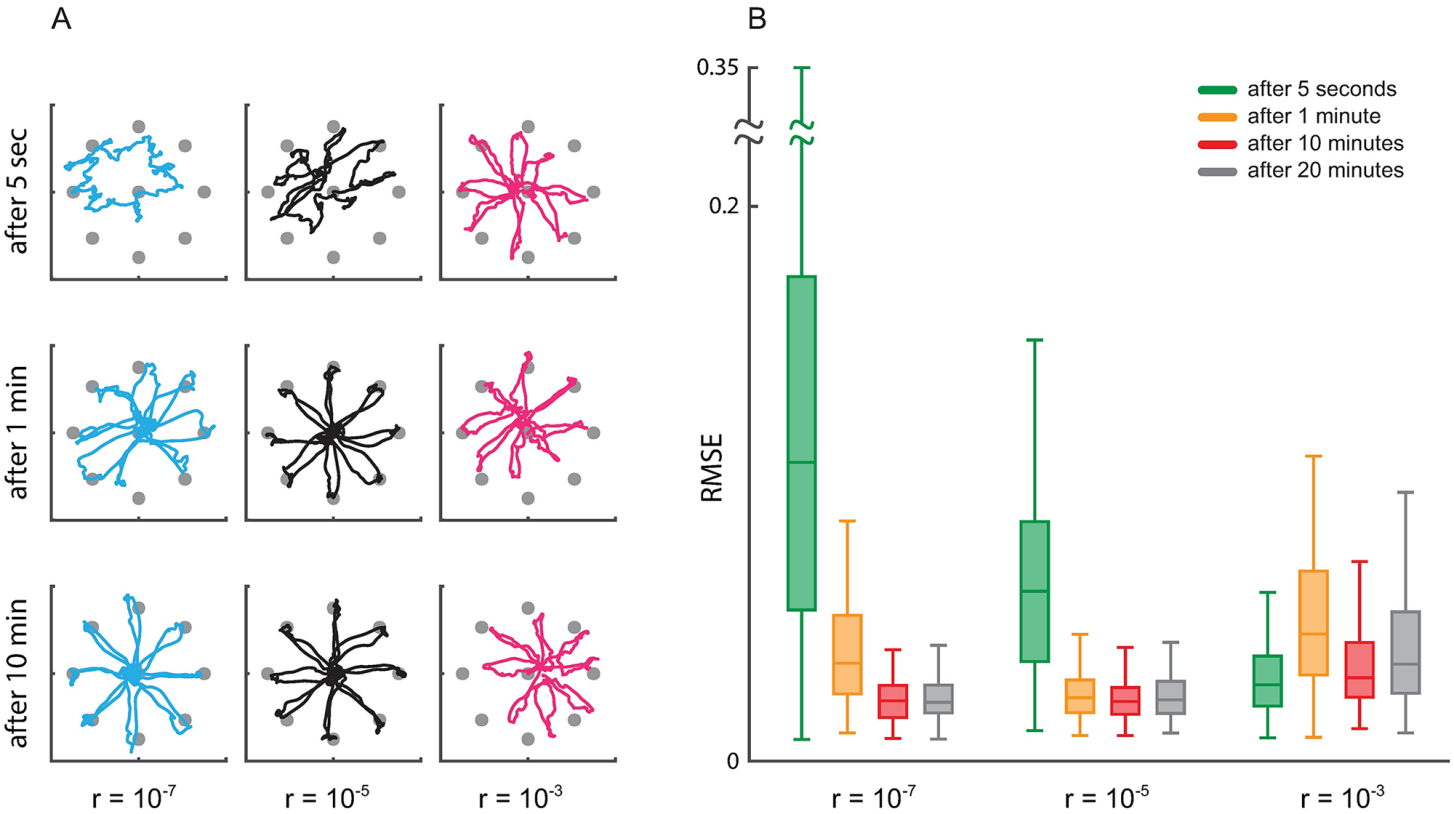
Learning rate calibration affects both the transient and the steady-state performance of closed-loop BMI decoders with discrete spiking activity. Figure conventions are the same as Fig 8. (A) The evolution of the decoded trajectory across time under different learning rates *r*. Each color corresponds to one learning rate. As in Fig 8, the decoder is fixed after a given adaptation time is completed (as noted on each row). The fixed decoder is then used to generate the displayed trajectories. The decoding performance is unstable when the learning rate is large (*r* = 10^−3^), i.e., the performance widely oscillates. (B) RMSE of the decoded trajectory under different learning rates for different adaptation times. RMSE is computed for a fixed decoder that was obtained by stopping the adaptation at various times (different colors). RMSE converges faster as the learning rate is increased (*r* = 10^−7^ to 10^−5^, for example). However, if the learning rate is selected too large (*r* = 10^−3^), RMSE oscillates without converging to a stable number. These results again demonstrate the importance of calibrating the learning rate for fast convergence and accuracy of decoding.

To study the effect of the learning rate on steady-state BMI decoding, we adaptively estimate the encoding model parameters under different learning rates. We fix the estimated parameters after varying amounts of adaptation time. We then use the obtained fixed models to run the closed-loop BMI simulations without adaptation. We run these simulations both for continuous LFP/ECoG observations decoded with a KF kinematic decoder, and for discrete spike observations decoded with a PPF kinematic decoder (Figs 8 and 9, respectively).

By comparing the small and medium learning rates, we find that a small learning rate results in a slow rate of convergence for the decoder performance, without improving the steady-state performance (two-sided t-test *P* > 0.36; Figs 8 and 9). Moreover, large learning rates result in poor and unstable steady-state decoding due to inaccurate estimation of the model parameters. This is evident by observing that for large learning rates, BMI decoding RMSE widely oscillates as a function of time at which adaptation stops for both continuous ECoG/LFP observations and discrete spike observations (Figs 8B and 9B, respectively). This result shows that due to the large steady-state error, steady-state parameter estimates change widely depending on exactly when we stop the adaptation. Thus the decoder does not converge to a stable performance. Taken together, optimally selecting the learning rate to achieve a desired level of steady-state parameter error covariance is also important for fast convergence and accuracy of decoding.

It is interesting to note that due to feedback-correction in closed-loop BMI, the decoder can tolerate a larger steady-state parameter error than we would typically allow if our only goal is to track the encoding model parameters. This is evident by noting, for example, that using a learning rate of *s* = 5 × 10^−3^ for continuous signals results in a relatively large steady-state parameter error as shown in Fig 4 (The 95% confidence bound is about ±30% of the modulation depth). However, for the purpose of BMI decoding, this learning rate results in no loss of performance at steady state compared to smaller learning rates, and allows for a faster convergence time (Fig 8). Hence the user-defined upper-bound on the steady-state error covariance is dependent on the application and the goal of adaptation. For closed-loop decoding, a larger error covariance could be tolerated, and as a result, a faster convergence time can be achieved. In contrast, if the goal is to accurately track the evolution of encoding models over time, for example to study learning and plasticity, a lower steady-state error covariance should be targeted. Regardless of the desired upper-bound on the error covariance, the calibration algorithm can closely approximate the corresponding learning rate that satisfies this upper-bound while allowing for the fastest possible convergence.

## Discussion

Developing invasive closed-loop neurotechnologies to treat various neurological disorders requires adaptively learning accurate encoding models that relate the recorded activity—whether in the form of spikes, LFP, or ECoG—to the underlying brain state. Fast and accurate adaptive learning of encoding models is critically affected by the choice of the learning rate [37], which introduces a fundamental trade-off between the steady-state error and the convergence time of the estimated model parameters. Despite the importance of the learning rate, currently a principled approach for its calibration is lacking. Here, we developed a principled analytical calibration algorithm for optimal selection of the learning rate in adaptive methods. We designed the calibration algorithm for two possible user-specified adaptation objectives, either to keep the parameter estimation error covariance smaller than a desired value while minimizing convergence time, or to keep the parameter convergence time faster than a given value while minimizing error. We also derived the calibration algorithm both for discrete-valued spikes modeled as point processes nonlinearly dependent on the brain state, and for continuous-valued neural recordings, such as LFP and ECoG, modeled as Gaussian processes linearly dependent on the brain state. We showed that the calibration algorithm allows for fast and accurate learning of encoding model parameters (Figs 4 and 7), and enables fast convergence of decoding performance and accurate steady-state decoding (Figs 8 and 9). We also demonstrated that larger learning rates make the encoding model and the decoding performance inaccurate, and smaller learning rates delay their convergence. The calibration algorithm provides an analytical approach to predict the effect of the learning rate in advance, and thus to select its optimal value prior to real-time adaptation in closed-loop neurotechnologies.

To derive the calibration algorithm, we introduced a formulation based on the fundamental trade-off that the learning rate dictates between the steady-state error and the convergence time of the estimated parameters. Calibrating the learning rate analytically requires deriving two functions that describe how the learning rate affects the convergence time and the steady-state error covariance, respectively. However, currently no explicit functions exist for these two relationships for Bayesian filters, such as the Kalman filter or the point process filter. We showed that the two functions can be analytically derived (Eqs (7), (8) and (19)) and can accurately predict the effect of the learning rate (Figs 3 and 6). We obtained the calibration algorithm by deriving two inverse functions that solve for the learning rate based on a given upper-bound of the error covariance (Eq (9)) or the convergence time (Eq (10)), respectively.

To allow for rigorous derivations in finding tractable analytical solutions for the learning rate, we performed the derivations for the case in which the behavioral state in the training experiment evolved periodically over time. This is the case in many applications; for example, in motor BMIs, models are often learned during a training session in which subjects perform a periodic center-out-and-back movement. However, we found that the calibration algorithm only depended on an average value of the behavioral state rather than on its periodic characteristics. Indeed, we showed that with a simplifying assumption, the derivation extends to the general non-periodic case (Appendix E in S1 Text, S2 Fig); moreover, using extensive numerical simulations, we demonstrated that the calibration algorithm can accurately predict the effect of the learning rate on parameter error and convergence time for a general behavioral state evolution in the training experiments (Figs 5 and 6B). The match between the analytical prediction of the calibration algorithm and the simulation results suggest the generalizability of the calibration algorithm across various behavioral state evolutions.

We derived the calibration algorithm for Bayesian adaptive filters, i.e., KF for continuous-valued activity and PPF for discrete-valued spikes. Here the KF and PPF were used to adaptively learn the neural encoding model parameters, which were assumed to be unknown but essentially fixed within the time-scales of parameter learning. This scenario is largely the case that arises in neurotechnologies for learning encoding models/decoders for two reasons. First, in neurotechnologies, such as BMIs, the parameters of the encoding models are initially unknown because they need to be learned in real time during closed-loop operation (cannot be learned offline and a-priori before actually using the BMI). Second, even though these parameters are unknown, they are largely fixed at least within relevant time-scales of parameter learning (e.g., minutes) in BMIs (and even typically within time-scales of BMI operation in a day, e.g., hours; see for example [17–19, 21–24, 26, 28, 29, 49–51, 57–64]). Even in scenarios where these parameters may change over time for example due to plasticity or task learning, the time-scale of parameter variation will be substantially slower than the time-scale of parameter estimation/learning in the KF or PPF. For example, as we show here and as observed in prior experiments through trial and error, with a well-calibrated adaptive algorithm the parameters can typically be learned within several minutes (e.g., [22–29]). In contrast, the time-scale of changes in encoding model parameters is typically on the order of days [18, 19, 56]. So even in the case that parameters may be changing, for the purpose of selecting the learning rate in the adaptive algorithm, they can be considered as essentially constant. We also showed that the calibration algorithm combined with the Bayesian adaptive filter can be used on an as-needed basis to re-learn parameters in case they shift over these relevant longer time-scales, e.g., from day to day. Finally, while Bayesian adaptive filters such as the KF and PPF can be used to track time-varying parameters, they can also be used to estimate fixed but unknown parameters as shown both in neurotechnologies and in other applications such as climate modeling, control of fluid dynamics, and robotics [28, 29, 65–69], and confirmed in our derivations and simulations here.

In deriving the calibration algorithm, we assumed that recorded signals (whether continuous or discrete) are conditionally independent over channels and in time, similar to prior work [17, 22, 23, 26–29, 49, 54–58, 61, 72]. This assumption enables the derivation of tractable real-time decoders (i.e., KF and PPF), adaptive algorithms, and in our case the analytical calibration algorithm, for both linear and nonlinear observation models (Eqs (1) and (12)) for continuous neural signals and binary spike events, respectively. While conditional dependencies could exist in general, prior experiments have shown that algorithms derived with these conditional independence assumptions work well for neural data analysis [17, 22, 23, 26–29, 49, 54–58, 61, 72]. Finally, given the high dimensionality of neural recordings obtained in current neurotechnologies, modeling correlations between channels would introduce a large number of unknown neural parameters that need to be learned in real time. This real-time learning becomes computationally quite expensive, and would require more data (and thus longer time in real-time applications) for parameters to be learned without overfitting. Thus the conditional independence assumption makes the parameter learning algorithms and setups amenable for real-time applications by reducing the number of model parameters and complexity.

The selected learning rate in the calibration algorithm depends on the user-specified upper-bound on the error covariance or convergence time. The values of these upper-bounds could be chosen by the user based on the goal of adaptation. If the adaptation goal is to accurately estimate the encoding model parameters (e.g., to study learning), then the acceptable error upper-bound may be selected to be small. In such a case, the calibration algorithm would select a small learning rate. However, we showed that if the goal of calibration is to enable accurate decoding in a closed-loop BMI, then larger errors in the estimated parameters may be tolerated. This is due to feedback-correction in BMIs, which can compensate for the parameter estimation error (Figs 8 and 9). The calibration algorithm would then select larger learning rates to improve how fast decoding performance converges to high values. However, even in this case, there is a limit to how large the learning rate can be. A learning rate that is too large will result in unstable and inaccurate performance of the decoder (Figs 8 and 9). This result shows the importance of the calibration algorithm regardless of the goal of adaptation.

The calibration algorithm may also serve as a tool to help examine the interaction between model adaptation and neural adaptation. In closed-loop neurotechnologies, neural representations can change over time resulting in neural adaptation, e.g., due to learning over multiple days. For example, in motor BMIs, the brain can change its encoding of movement (e.g., the directional tuning of neurons) to improve neuroprosthetic control [17–19, 56, 85]. Neural and model adaptation result in a “two-learner system” and can interact [56]. It is important to study whether model adaptation interferes with neural adaptation in these closed-loop systems, and if so whether this interference depends on how fast models are adapted. By accurately adjusting the convergence time and hence the speed of model adaptation, the calibration algorithm may provide a useful tool in studying such interference in careful experiments. Moreover, if neural adaptation is significantly affected by the speed of model adaptation, the calibration algorithm could help carefully adjust this speed for a desired neural adaptation outcome. It is also important to examine this interference problem theoretically [86].

To validate the calibration algorithm, we used a motor BMI as an example. The calibration algorithm, however, can be applied to other closed-loop neurotechnologies that need to decode various brain states, for example, interest score in closed-loop cortically-coupled computer vision for image search [87] or mood in closed-loop DBS systems [88]. Also, while our main goal was to derive the calibration algorithm for closed-loop neurotechnologies, this algorithm can be used in other domains of signal processing. We derived the calibration algorithm to select the learning rate and predict its effect on error and convergence time in Bayesian adaptive filters. Prior work in other signal processing applications have focused vastly on the non-Bayesian LMS or steepest-decent adaptive filters [37, 40]. However, LMS is only applicable to linear observation models [37]. Moreover, steepest-decent filters that use non-linear cost functions to specify the goal of adaptation cannot predict the effect of learning rate on error or convergence time and thus only provide heuristics for learning rate selection [37]. Finally, LMS or steepest-decent filters are not Bayesian filters, unlike the KF or the PPF (Eqs (3)–(6) and (15)–(18)). Using a Bayesian filter for parameter adaptation has the advantage that it can extend to nonlinear stochastic observation models (such as the point process model of spikes) [28, 29, 36]. Here, we derived a learning rate calibration algorithm for Bayesian filters both with continuous linear observation models (KF) and with discrete nonlinear observations models (PPF). Importantly, we derived explicit analytical functions (9) and (10) to predict the effect of the learning rate on steady-state error and convergence time for a Bayesian filter. This allowed us to analytically compute an optimal value for the learning rate to achieve a desired user-specified performance metric.

Our main contribution is the derivation of a novel analytical calibration algorithm for both nonlinear point process and linear Gaussian encoding models (Eqs (1) and (12)); this calibration algorithm optimally selects the learning rate based on the trade-off between convergence time and steady-state error covariance. In deriving closed-form expressions for the calibration algorithm, we needed to *analytically* compute the steady-state error covariance in both the PPF and the KF. Note that, even in the case of the KF, this analytical computation cannot be achieved through the general steady-state analysis of the KF. First, the steady-state analysis of the KF does not formulate a tradeoff between the steady-state error covariance and convergence time, and thus does not provide a calibration algorithm. Second, in order to derive the calibration algorithm, we need to derive novel *analytical closed-form* expressions for the steady-state error covariance and convergence time in the KF (so that we can find the inverse function to compute the optimal learning rate for a given covariance or convergence time). To obtain these expressions, we need to find an analytical solution for a special form of the discrete Riccati equation (DRE) [89]. While the DRE is solved numerically and recursively in the general steady-state analysis of a KF, there exists no analytical solution with a closed-form expression for a DRE in general. Obtaining such an analytical solution is critical for calculating the optimal learning rate in (9) and (10). Therefore, unlike the steady-state analysis of KF, we additionally had to derive the analytic solution of a special form of DRE first (Appendix J in S1 Text). Third, we also needed analytical expressions for the convergence time of the KF during the transient phase, which again the steady-state analysis of the KF does not provide. Finally, note that we also provide the calibration algorithm for the point process model of the binary spike time-series and thus for the nonlinear PPF in addition to the linear KF.

Here our focus was on deriving an analytical calibration algorithm for both nonlinear point process and linear Gaussian encoding models for spikes and continuous neural recordings, respectively. Thus to validate our analytical approach, we used extensive closed-loop Monte-Carlo simulations. These simulations allowed us to examine the generalizability of the calibration algorithm across different neural signal modalities. The closed-loop simulations closely conformed to our prior non-human primate experiments [28, 29]. Prior studies have shown that these closed-loop simulations can mimic the observed experimental effects and thus provide a useful validation testbed for algorithms [28, 38, 48, 90]. Moreover, the calibration algorithm adjusted the learning rate of adaptive PPF and adaptive KF decoders, which have been shown to be successful for real-time BMI training and control using spikes or LFP in non-human primate and human experiments both in our work and other studies [17, 21–30, 55]. However, prior experiments, including ours, selected the learning rates empirically in these decoders. Given that the calibration algorithm is run prior to experiments, and based on the success of adaptive PPF and KF in prior animal and human experiments, we expect our calibration algorithm to be seamlessly incorporated in BMIs regardless of the neural signal modality. The calibration algorithm allows the optimal learning rate to be computed prior to running the adaptation experiments to achieve a predictable speed and accuracy in adaptive learning. Implementing the calibration algorithm in animal models of adaptive BMIs using both spikes and LFP is the topic of our future investigation.

Finally, the calibration algorithm has the potential to be generalized to Bayesian filters beyond the KF and PPF, e.g., the unscented Kalman filter [42], an adaptive filter with a binomial distribution as the observation model [44], or hybrid spike-LFP filters [91]. The derivations of Eqs (7) and (8) in theorems 1 and 3 are based on the recursive equation for estimation error dynamics, which is derived from the desired Bayesian filter. This implies that for other observation models different from a linear model with Gaussian noise in KF or a nonlinear point process model in PPF, once we write down their corresponding Bayesian adaptive filters [92], we can derive the calibration algorithms by writing the corresponding recursive error equations. Thus this calibration algorithm has the potential to be generalized and applied to other types of signals with various stochastic models. This will be a topic of our future investigation.

## Supporting information

S1 FigThe calibration algorithm along with the recursive Bayesian decoder can be used on an as-needed basis to re-learn encoding models as parameters shift over time.Simulation of a BMI system in which parameters are estimated at the beginning of each day and fixed for the rest of the day. This is the setup used in the vast majority of BMI systems because encoding model parameters are either largely time-invariant or change much slower compared with the relevant time-scales of parameter adaptive learning (e.g., minutes) in BMIs and even the time-scale of BMI operation in a day (e.g., hours) (see Discussion). Figure convention is the same as in Fig 4. Here we show the example of the KF whose learning rate is selected using the calibration algorithm to satisfy user-specified criteria on steady-state error and convergence time as described in Results and shown in Fig 4B. As the task is the same on both days and since **H**_*ave*_ is simply an expectation (average) of a function of ${\tilde{v}}_{t}$ and does not need knowledge of ${\tilde{v}}_{t}$ values, we used the same **H**_*ave*_ based on the same average quantity to compute the optimal learning rate on both days. The calibration algorithm satisfies the user-specified criteria on parameter estimates on day 1. We then assume that on day 2 parameters have shifted. On day 2, parameters can again be estimated using the same Kalman filter whose learning rate is selected with the calibration algorithm. Similar to day 1, on day 2 the requirements on steady-state error and convergence time are again satisfied.(TIF)

S2 Fig Sketch of the derivation of the calibration algorithm. The derivation of the calibration algorithm with a periodic encoded state **v**_*t*_ during the training session follows the blue arrows. If we assume that the prediction covariance **S**_*t*+1|*t*_ has bounded steady-state moments, then the proof generalizes to the non-periodic **v**_*t*_ as shown by the red arrows (see Appendix E in S1 Text and Fig 5). Similarly for the PPF, if we assume that the prediction covariance **Q**_*t*+1|*t*_ has bounded steady-state moments, then the proof generalizes to the non-periodic **v**_*t*_ (Fig 6B) and the mean of **Q**_*t*+1|*t*_ at steady state can be approximated using **M**_*ave*_ in Theorem 3 to find the optimal learning rate. Here DRE refers to the discrete Riccati equation.
(TIF)

S1 Text All appendixes (A–J). (PDF)
