# Supplementary figures and images for: Optimizing the learning rate for adaptive estimation of neural encoding models
Source: PLoS Comput Biol. 2018 May 29;14(5):e1006168. doi: 10.1371/journal.pcbi.1006168 (PMC5993334; doi:10.1371/journal.pcbi.1006168)

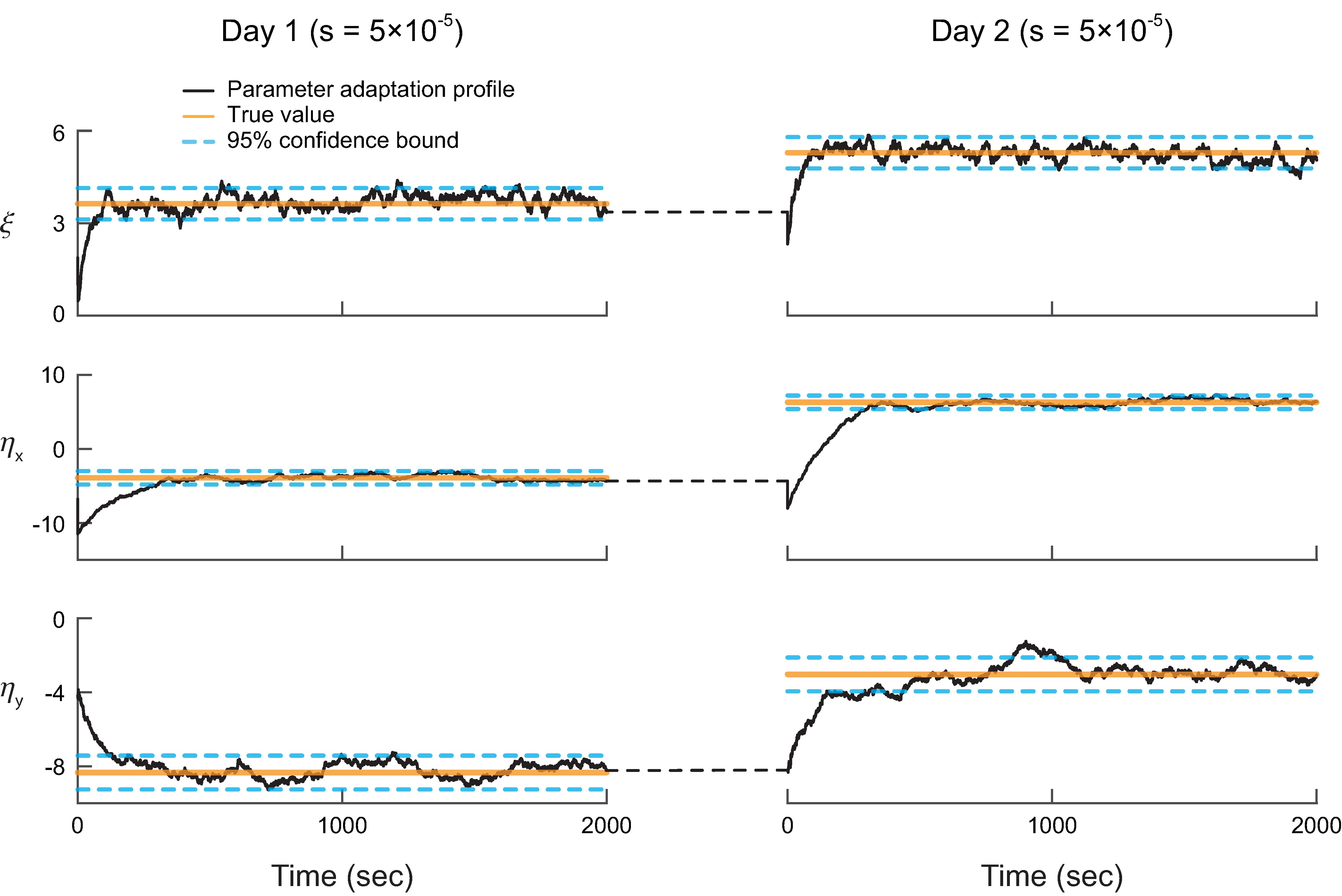

Supplement: S1 Fig — Simulation of a BMI system in which parameters are estimated at the beginning of each day and fixed for the rest of the day. This is the setup used in the vast majority of BMI systems because encoding model parameters are either largely time-invariant or change much slower compared with the relevant time-scales of parameter adaptive learning (e.g., minutes) in BMIs and even the time-scale of BMI operation in a day (e.g., hours) (see Discussion). Figure convention is the same as in Fig 4. Here we show the example of the KF whose learning rate is selected using the calibration algorithm to satisfy user-specified criteria on steady-state error and convergence time as described in Results and shown in Fig 4B. As the task is the same on both days and since Have is simply an expectation (average) of a function of v˜t and does not need knowledge of v˜t values, we used the same Have based on the same average quantity to compute the optimal learning rate on both days. The calibration algorithm satisfies the user-specified criteria on parameter estimates on day 1. We then assume that on day 2 parameters have shifted. On day 2, parameters can again be estimated using the same Kalman filter whose learning rate is selected with the calibration algorithm. Similar to day 1, on day 2 the requirements on steady-state error and convergence time are again satisfied. (TIF) [file pcbi.1006168.s001.tif]

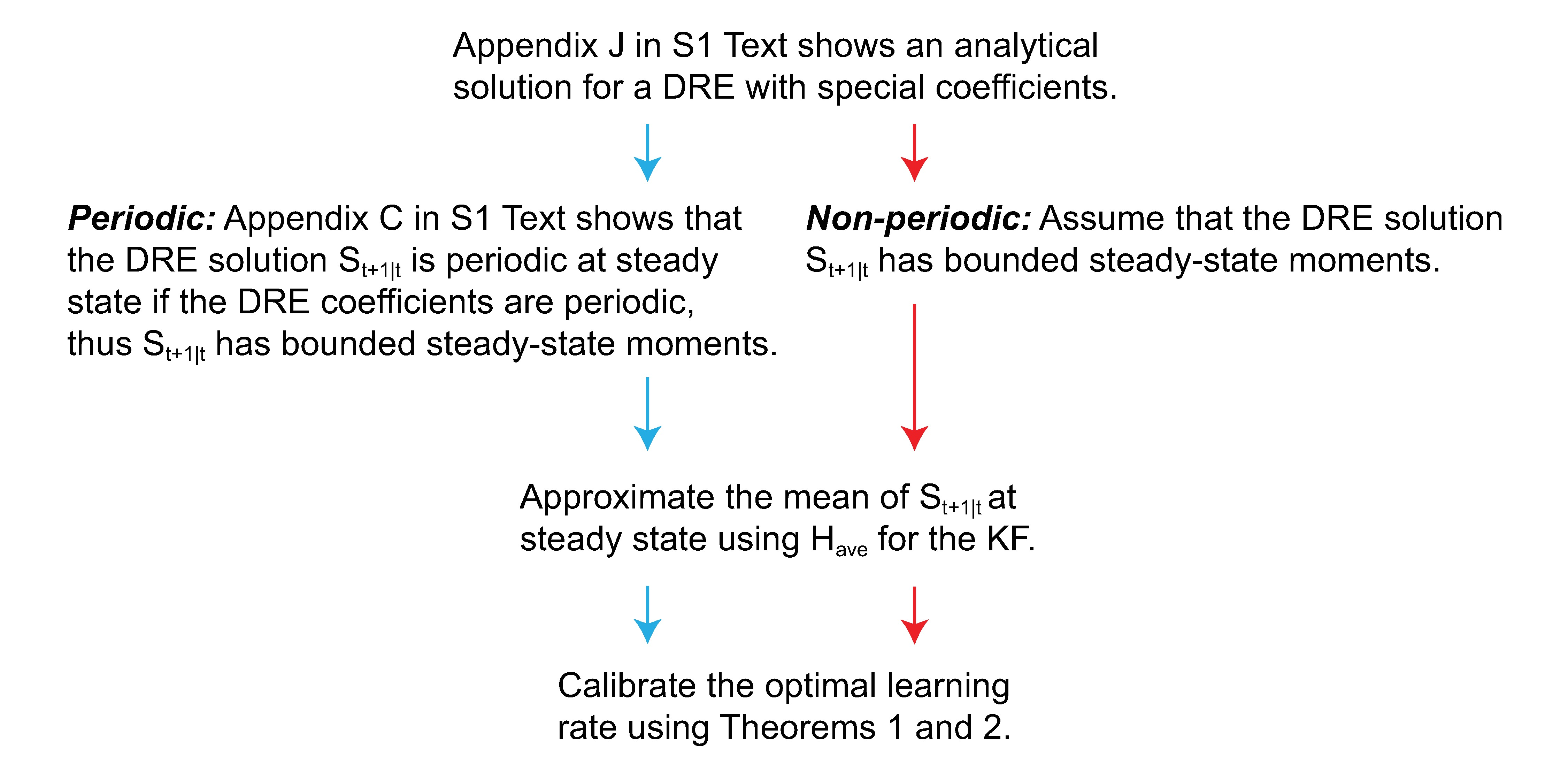

Supplement: S2 Fig — The derivation of the calibration algorithm with a periodic encoded state vt during the training session follows the blue arrows. If we assume that the prediction covariance St+1|t has bounded steady-state moments, then the proof generalizes to the non-periodic vt as shown by the red arrows (see Appendix E in S1 Text and Fig 5). Similarly for the PPF, if we assume that the prediction covariance Qt+1|t has bounded steady-state moments, then the proof generalizes to the non-periodic vt (Fig 6B) and the mean of Qt+1|t at steady state can be approximated using Mave in Theorem 3 to find the optimal learning rate. Here DRE refers to the discrete Riccati equation. (TIF) [file pcbi.1006168.s002.tif]
