# Supplementary material for: Optimizing the learning rate for adaptive estimation of neural encoding models
Source: PLoS Comput Biol. 2018 May 29;14(5):e1006168. doi: 10.1371/journal.pcbi.1006168 (PMC5993334; doi:10.1371/journal.pcbi.1006168)
Supplement: S1 Text — (PDF) [file pcbi.1006168.s003.pdf]

S1 Text. All appendixes (A–J)

Appendix A: The Role of s and r in Prior Models of Adaptive KF/PPF

Here we clarify why s in the covariance matrix $\mathbf{S} = s\mathbf{I}_n$ in (2) for the adaptive KF and r in the covariance matrix $\mathbf{Q} = r\mathbf{I}_n$ in (14) for the adaptive PPF specify the learning rates. For the KF, the covariance matrix $\mathbf{S} = s\mathbf{I}_n$ in (2) models our uncertainty about the unknown underlying parameters $\boldsymbol{\psi}$. This indeed implies that s also has a critical role for parameter estimation: it balances the relative weight of the previous parameter estimate $\boldsymbol{\psi}_{t-1|t-1}$ and the latest observation y_t in obtaining the new parameter estimate $\boldsymbol{\psi}_{t|t}$ in the KF. By doing so, s controls how fast parameters are learned. This is the reason that we call s the learning rate. Similarly, r plays the same role for the PPF.

To see the above more explicitly, we can combine equations (3) and (6) for the adaptive KF. This gives the following update equation for the KF:

$$\boldsymbol{\psi}_{t|t} = \boldsymbol{\psi}_{t-1|t-1} + \mathbf{S}_{t|t}\tilde{\mathbf{v}}_t Z^{-1}(y_t - \tilde{\mathbf{v}}_t'\boldsymbol{\psi}_{t-1|t-1}). \tag{A1}$$

Similarly, by combining equations (15) and (18) for the adaptive PPF, we get the following update equation

$$\boldsymbol{\phi}_{t|t} = \boldsymbol{\phi}_{t-1|t-1} + \mathbf{Q}_{t|t}\tilde{\mathbf{v}}_t[N_t - \lambda(t|\boldsymbol{\phi}_{t-1|t-1})\Delta]. \tag{A2}$$

The relative weight of the neural observation y_t and the previous estimated parameters $\boldsymbol{\psi}_{t-1|t-1}$ in the KF is governed by $\mathbf{S}_{t|t}\tilde{\mathbf{v}}_t Z^{-1}$ in (A1). The only element of the relative weight in (A1) that is in our control is $\mathbf{S}_{t|t}$. From equation (A21) in Appendix D, the eigenvalues $\{\kappa_m\}$ of the steady-state average of $\mathbf{S}_{t|t}$, which is denoted by $\tilde{\mathbf{S}}_+$, can be written as

$$\begin{aligned}\kappa_m &= \frac{\sqrt{h_m^2 s^2 + 4h_m s} - h_m s}{2h_m} \\ &= \frac{2}{\sqrt{h_m^2 + 4h_m/s} + h_m}.\end{aligned} \tag{A3}$$

These eigenvalues are thus a monotonically increasing function of s (note that h_m is a constant). So as s increases, the eigenvalues of $\mathbf{S}_{t|t}$ increase and thus the parameters are learned faster but at the price of a higher steady-state variation and error (theorem 1), and vice versa. That is why we refer to s as the learning rate because it controls the rate of parameter update as in equation (A1).

Similarly, in the PPF, the relative weight of the neural observation N_t and the previous estimated parameters $\boldsymbol{\phi}_{t-1|t-1}$ is governed by $\mathbf{Q}_{t|t}\tilde{\mathbf{v}}_t$ in (A2). The only factor that is in our control in the relative weight in equation (A2) is $\mathbf{Q}_{t|t}$, which is in turn purely controlled by the design parameter r. Again, from Appendix H, the eigenvalues $\{b_m\}$ of the steady-state average of $\mathbf{Q}_{t|t}$, which is denoted by $\tilde{\mathbf{Q}}_+$, can be written as

$$\begin{aligned}b_m &= \frac{\sqrt{a_m^2 r^2 + 4a_m r} - a_m r}{2a_m} \\ &= \frac{2}{\sqrt{a_m^2 + 4a_m/r} + a_m}.\end{aligned} \tag{A4}$$

These eigenvalues are thus a monotonically increasing function of r (note that a_m is a constant). So as r increases, parameters are learned faster, which is why we refer to r as the learning rate for the PPF.

Appendix B: KF and PPF for Kinematic Decoding in Closed-loop BMIs

Here we present the kinematic decoders, which use the learned encoding models. We use a KF for continuous signals and a PPF for discrete spikes. In both decoders, the prior model on the kinematic state $\mathbf{x}_t = [\mathbf{d}_t', \mathbf{v}_t']'$ is given as [1–6]

$$\mathbf{x}_{t+1} = \mathbf{A}\mathbf{x}_t + \mathbf{w}_t, \tag{A5}$$

where $\mathbf{w}_t$ is a white Gaussian noise with covariance matrix $\mathbf{W}$ and

$$\mathbf{A} = \begin{bmatrix} 1 & 0 & \Delta & 0 \\ 0 & 1 & 0 & \Delta \\ 0 & 0 & \alpha & 0 \\ 0 & 0 & 0 & \alpha \end{bmatrix} \tag{A6}$$

with α enforcing correlation between consecutive velocities and thus continuity in the evolution of kinematics. For observations, we assume that there are C channels.

For continuous neural signals, the observation model is given by combining all channels described in (1) into a single vector equation as follows

$$\mathbf{y}_t = \boldsymbol{\xi} + \mathbf{H}\mathbf{x}_t + \mathbf{z}_t \tag{A7}$$

where $\mathbf{y}_t = [y_t^1, ..., y_t^C]'$, $\boldsymbol{\xi} = [\xi^1, ..., \xi^C]'$ is the baseline activity, $\mathbf{z}_t = [z_t^1, ..., z_t^C]'$ is the zero-mean white Gaussian noise, and the c-th row of $\mathbf{H}$ is $[0, 0, \eta_x^c, \eta_y^c] = [\mathbf{0}', \boldsymbol{\eta}^{c\prime}]$. Equations (A5) and (A7) form a linear state-space model. Denoting the posterior and prediction means by $\mathbf{x}_{t|t}$ and $\mathbf{x}_{t|t-1}$, and their covariances by $\mathbf{U}_{t|t}$ and $\mathbf{U}_{t|t-1}$, respectively, the kinematic state, $\mathbf{x}_t$, can be decoded by KF as follows [7]

$$\mathbf{x}_{t|t-1} = \mathbf{A}\mathbf{x}_{t-1|t-1} \tag{A8}$$

$$\mathbf{U}_{t|t-1} = \mathbf{A}\mathbf{U}_{t-1|t-1}\mathbf{A}' + \mathbf{W} \tag{A9}$$

$$\mathbf{U}_{t|t}^{-1} = \mathbf{U}_{t|t-1}^{-1} + \mathbf{H}'\mathbf{Z}^{-1}\mathbf{H} \tag{A10}$$

$$\mathbf{x}_{t|t} = \mathbf{x}_{t|t-1} + \mathbf{U}_{t|t}\mathbf{H}'\mathbf{Z}^{-1}\big[\mathbf{y}_t - \boldsymbol{\xi} - \mathbf{H}\mathbf{x}_{t|t-1}\big] \tag{A11}$$

For discrete observations (i.e., spikes), the prior kinematic model for $\mathbf{x}_t$ is the same as (A5). We denote $N_t^{1:C} = [N_t^1, ..., N_t^C]'$. Given the assumption that neurons are conditionally independent conditioned on $\mathbf{x}_t$ as before, the observation model can be written down using (12) as

$$\begin{aligned} p(N_t^{1:C}|\mathbf{x}_t) &= \prod_{c=1}^{C} p(N_t^c|\mathbf{x}_t) \\ &= \prod_{c=1}^{C} (\lambda^c(\mathbf{x}_t)\Delta)^{N_t^c} e^{-\lambda^c(\mathbf{x}_t)\Delta} \end{aligned} \tag{A12}$$

Here $\lambda^c(\mathbf{x}_t) = \lambda^c(\mathbf{v}_t) = \exp(\beta^c + (\boldsymbol{\alpha}^c)'\mathbf{v}_t)$. This is well-defined because $\mathbf{x}_t = [\mathbf{d}_t', \mathbf{v}_t']'$. From (A5) and (A12), the kinematic state $\mathbf{x}_t$ can be decoded by PPF as follows

$$\mathbf{x}_{t|t-1} = \mathbf{A}\mathbf{x}_{t-1|t-1} \tag{A13}$$

$$\mathbf{U}_{t|t-1} = \mathbf{A}\mathbf{U}_{t-1|t-1}\mathbf{A}' + \mathbf{W} \tag{A14}$$

$$\mathbf{U}_{t|t}^{-1} = \mathbf{U}_{t|t-1}^{-1} + \sum_{c=1}^{C} \tilde{\boldsymbol{\alpha}}^c(\tilde{\boldsymbol{\alpha}}^c)'\lambda^c(\mathbf{x}_{t|t-1})\Delta \tag{A15}$$

$$\mathbf{x}_{t|t} = \mathbf{x}_{t|t-1} + \mathbf{U}_{t|t} \times \sum_{c=1}^{C} \tilde{\boldsymbol{\alpha}}^c\big[N_t^c - \lambda^c(\mathbf{x}_{t|t-1})\Delta\big] \tag{A16}$$

where $\tilde{\boldsymbol{\alpha}}^c = [\mathbf{0}', (\boldsymbol{\alpha}^c)']'$. The derivation details can be found in [4, 6].

Appendix C: Unbiased Estimation and Convergence of the Error Covariance Matrix

Here we show that the estimated parameters $\{\boldsymbol{\psi}_{t|t}\}$ in KF are asymptotically unbiased. We also show the condition for the covariance matrices $\mathbf{S}_{t|t-1}$ and $\mathbf{S}_{t|t}$ to converge to symmetric, periodic and positive definite (SPPD) solutions. We know from (4) and (5) that $\mathbf{S}_{t|t-1}$ and $\mathbf{S}_{t|t}$ satisfy the following jointly recursive functions

$$\begin{aligned}\mathbf{S}_{t|t-1} &= \mathbf{S}_{t-1|t-1} + \mathbf{S} \\ \mathbf{S}_{t|t}^{-1} &= \mathbf{S}_{t|t-1}^{-1} + \tilde{\mathbf{v}}_t \tilde{\mathbf{v}}_t' Z^{-1}\end{aligned}$$

where $\mathbf{S} = s\mathbf{I}_n$. From these two equations, it's well known that $\mathbf{S}_{t|t-1}$ can be calculated by the discrete Riccati equation (DRE) recursively [8]. This equation is given by

$$\mathbf{S}_{t+1|t} = \mathbf{S}_{t|t-1} + \mathbf{S} - \frac{\mathbf{S}_{t|t-1}\tilde{\mathbf{v}}_t \tilde{\mathbf{v}}_t' \mathbf{S}_{t|t-1}}{k_t}, \tag{A17}$$

where $k_t = Z + \tilde{\mathbf{v}}_t' \mathbf{S}_{t|t-1} \tilde{\mathbf{v}}_t$ is a time-dependent scalar. Generally, $\{\tilde{\mathbf{v}}_t\}$ can be any arbitrary time-varying state. If $\{\tilde{\mathbf{v}}_t\}$ is periodic, then (A17) becomes a discrete periodic Riccati equation (DPRE). Now from Theorem 6.12 in [9], we conclude that $\{\mathbf{S}_{t|t-1}\}$ converges toward a SPPD solution, which is the unique stabilizing solution of the DPRE and is independent from the initial condition $\mathbf{S}_{1|0}$. This is because the two conditions required in this theorem are met in our case (the pair $(\mathbf{I}_n, \sqrt{s}\,\mathbf{I}_n)$ is observable since $s > 0$ and the pair $(\mathbf{I}_n, \{\tilde{\mathbf{v}}_t\})$ is stabilizable since the behavioral state $\{\tilde{\mathbf{v}}_t\}$ in a well-designed training set explores the dynamic range of possible values.)

Also, from theorem 6.11 in [9], the gain matrix of $\boldsymbol{\psi}^* - \boldsymbol{\psi}_{t|t}$ is stable so $\{\boldsymbol{\psi}_{t|t}\}$ is asymptotically unbiased.

Appendix D: The Derivation of the Steady-State Error Covariance and the Convergence Time as Functions of the Learning Rate s in KF

Here we derive the analytic functions of the steady-state error covariance $\mathbf{S}^*_+$ and the convergence rate of $\mathrm{E}[\mathbf{g}_t]$ in (7) and (8), respectively. We derive $\mathbf{S}^*_+$ first. In the regular KF and when the parameters truly follow random-walk dynamics as given in (2), then for the parameter error covariance at time t, $\mathbf{S}^*_{t|t}$, we have $\lim_{t\to\infty} \|\mathbf{S}^*_{t|t} - \mathbf{S}_{t|t}\| = 0$. However, here the true parameter $\boldsymbol{\psi}^*$ is an unknown constant vector, which does not obey the random-walk model; indeed the random-walk model is simply used to approximate our uncertainty about the parameters. Due to this modeling mismatch, $\mathbf{S}^*_{t|t} < \mathbf{S}_{t|t}$ and $\lim_{t\to\infty} \|\mathbf{S}^*_{t|t} - \mathbf{S}_{t|t}\| \neq 0$. In other words, the KF is overestimating the error covariance because of model mismatch. Hence we need to estimate the difference.

We can derive the error dynamics, i.e., the dynamics of $\mathbf{g}_t$, from (6) as

$$
\begin{aligned}
\mathbf{g}_t &= \boldsymbol{\psi}^* - \boldsymbol{\psi}_{t|t} \\
&= \boldsymbol{\psi}^* - \boldsymbol{\psi}_{t|t-1} - \mathbf{S}_{t|t}\tilde{\mathbf{v}}_t Z^{-1}(y_t - \tilde{\mathbf{v}}_t'\boldsymbol{\psi}_{t|t-1}) \\
&= \boldsymbol{\psi}^* - \boldsymbol{\psi}_{t-1|t-1} - \mathbf{S}_{t|t}\tilde{\mathbf{v}}_t Z^{-1}(y_t - \tilde{\mathbf{v}}_t'\boldsymbol{\psi}_{t-1|t-1}) \\
&= \mathbf{g}_{t-1} - \mathbf{S}_{t|t}\tilde{\mathbf{v}}_t Z^{-1}(\tilde{\mathbf{v}}_t'\boldsymbol{\psi}^* + z_t - \tilde{\mathbf{v}}_t'\boldsymbol{\psi}_{t-1|t-1}) \\
&= \mathbf{g}_{t-1} - \mathbf{S}_{t|t}\tilde{\mathbf{v}}_t Z^{-1}(\tilde{\mathbf{v}}_t'\mathbf{g}_{t-1} + z_t) \\
&= (\mathbf{I} - \mathbf{K}_t\tilde{\mathbf{v}}_t')\mathbf{g}_{t-1} - \mathbf{K}_t z_t, \qquad \text{(A18)}
\end{aligned}
$$

where $\mathbf{K}_t = \mathbf{S}_{t|t}\tilde{\mathbf{v}}_t Z^{-1}$ and $\mathbf{I} - \mathbf{K}_t\tilde{\mathbf{v}}_t' = \mathbf{I} - \mathbf{S}_{t|t}\tilde{\mathbf{v}}_t Z^{-1}\tilde{\mathbf{v}}_t' = \mathbf{I} - \mathbf{S}_{t|t}\tilde{\mathbf{v}}_t\tilde{\mathbf{v}}_t' Z^{-1} = \mathbf{S}_{t|t}\mathbf{S}^{-1}_{t|t-1}$ from (5). The first line gives the second line by using (6), the second line gives the third line by using (3), and the third line gives the fourth line by using (1).

As we will see, the average value of the KF posterior covariance $\mathbf{S}_{t|t}$ and prediction covariance $\mathbf{S}_{t|t-1}$—which converge to symmetric, periodic, and positive definite (SPPD) solutions under mild conditions provided in Appendix C—are critical in deriving the steady-state error covariance $\mathbf{S}^*_+$ and the convergence time of $\mathrm{E}[\mathbf{g}_t]$. Hence (A18) will be used in the derivation of the calibration algorithm under both objectives in the subsequent sections.

Analytical Function for the Steady-State Error Covariance

Here we focus on the first objective for the calibration algorithm—keeping the steady-state error covariance under an upper-bound while minimizing the convergence time. The strategy for solving the steady-state error covariance $\mathbf{S}^*_+$ is to calculate the average of the posterior covariance $\mathbf{S}_{t|t}$ at steady state, denoted by $\widetilde{\mathbf{S}}_+$, and find the difference $(\widetilde{\mathbf{S}}_+ - \mathbf{S}^*_+)$. Since $\mathbf{S}_{t|t}$ and $\mathbf{S}_{t|t-1}$ would converge to a periodic solution from Appendix C, we can take their average. We first find the average values of $\mathbf{S}_{t|t}$ and $\mathbf{S}_{t|t-1}$, which are denoted by $\widetilde{\mathbf{S}}_+$ and $\widetilde{\mathbf{S}}_-$, at steady state respectively.

Here to make the derivation rigorous, we assume that the encoded state $\mathbf{v}_t$ is periodic with period T, as in theorem 1—as mentioned above, this requirement on $\mathbf{v}_t$ guarantees that the average steady-state values of the covariance matrices $\mathbf{S}_{t|t}$ and $\mathbf{S}_{t|t-1}$ exist because in this case these matrices will converge to a periodic solution and will be bounded (Appendix C). Let's denote by t^* the time after which $\mathbf{S}_{t|t}$ and $\mathbf{S}_{t|t-1}$ converge. We can compute the average steady-state posterior and prediction covariances $\widetilde{\mathbf{S}}_+$ and $\widetilde{\mathbf{S}}_-$ as

$$
\begin{aligned}
\widetilde{\mathbf{S}}_+ &= \frac{1}{T}\sum_{t=t^*+1}^{t^*+T} \mathbf{S}_{t|t} \\
\widetilde{\mathbf{S}}_- &= \frac{1}{T}\sum_{t=t^*+1}^{t^*+T} \mathbf{S}_{t|t-1}.
\end{aligned}
$$

We take an average over both sides of (4) and (5). Doing so we have

$$\widetilde{\mathbf{S}}_- = \widetilde{\mathbf{S}}_+ + \mathbf{S} \tag{A19}$$

$$\widetilde{\mathbf{S}}_+^{-1} = \widetilde{\mathbf{S}}_-^{-1} + \mathbf{H}_{ave} \tag{A20}$$

where $\mathbf{S} = s\mathbf{I}$ and $\mathbf{H}_{ave} = \frac{1}{T}\sum_{t=t^*+1}^{t^*+T} \tilde{\mathbf{v}}_t\tilde{\mathbf{v}}_t' Z^{-1} = \frac{1}{T}\sum_{t=1}^{T} \tilde{\mathbf{v}}_t\tilde{\mathbf{v}}_t' Z^{-1}$. We get the last equality because $\mathbf{v}_t$ is periodic with period T, so we can shift the index in the summation. Note that in taking the average on (5), we make an approximation by interchanging the order of matrix inversion and the average operator to get (A20). As we prove in Appendix I, this interchange is reasonable for a small learning rate s since in this case

$$(\frac{1}{T}\sum_{t=t^*+1}^{t^*+T} \mathbf{S}_{t|t})^{-1} - \frac{1}{T}\sum_{t=t^*+1}^{t^*+T} \mathbf{S}_{t|t}^{-1} \approx (\frac{1}{T}\sum_{t=t^*+1}^{t^*+T} \mathbf{S}_{t|t-1})^{-1} - \frac{1}{T}\sum_{t=t^*+1}^{t^*+T} \mathbf{S}_{t|t-1}^{-1}.$$

We now apply the lemma in Appendix J to calculate $\widetilde{\mathbf{S}}_+$ as an explicit function of the learning rate s. Using the Eigenvalue decomposition, we write $\mathbf{H}_{ave} = \mathbf{U}\ diag(h_1, ..., h_n)\ \mathbf{U}'$ $(0 < h_i \leq h_{i+1})$ where $\mathbf{U}$ is a unitary matrix. Then $\widetilde{\mathbf{S}}_+ = \mathbf{U}\ diag(\kappa_1, ..., \kappa_n)\ \mathbf{U}'$ and $(m = 1, ..., n)$

$$\kappa_m = \frac{\sqrt{h_m^2 s^2 + 4h_m s} - h_m s}{2h_m} \tag{A21}$$

$$h_m = \frac{1}{\kappa_m} - \frac{1}{\kappa_m + s}. \tag{A22}$$

Since $\widetilde{\mathbf{S}}_+$ can be expressed as a function of the learning rate s and $\mathbf{H}_{ave}$, we only need $\widetilde{\mathbf{S}}_+ - \mathbf{S}_+^*$ to find the steady-state error covariance $\mathbf{S}_+^*$.

We can now solve for the steady-state error covariance $\mathbf{S}_+^*$ from (A18). The estimation error covariance $\mathbf{S}_{t|t}^*$ can be calculated recursively by taking the variance of both sides of (A18). This recursive equation is given in (A23). For calculating the difference between $\widetilde{\mathbf{S}}_+$ and $\mathbf{S}_+^*$, which are the values of $\mathbf{S}_{t|t}$ and $\mathbf{S}_{t|t}^*$ at steady state, we also write the recursive equation for $\mathbf{S}_{t|t}$ below, which is in the Joseph form [10]. This recursion is given in (A24).

$$\mathbf{S}_{t|t}^* = (\mathbf{I} - \mathbf{K}_t\tilde{\mathbf{v}}_t')\mathbf{S}_{t-1|t-1}^*(\mathbf{I} - \mathbf{K}_t\tilde{\mathbf{v}}_t')' + \mathbf{K}_t Z\mathbf{K}_t' \tag{A23}$$

$$\mathbf{S}_{t|t} = (\mathbf{I} - \mathbf{K}_t\tilde{\mathbf{v}}_t')\mathbf{S}_{t-1|t-1}(\mathbf{I} - \mathbf{K}_t\tilde{\mathbf{v}}_t')' + \mathbf{K}_t Z\mathbf{K}_t' + (\mathbf{I} - \mathbf{K}_t\tilde{\mathbf{v}}_t')\mathbf{S}(\mathbf{I} - \mathbf{K}_t\tilde{\mathbf{v}}_t')' \tag{A24}$$

Subtracting (A23) from (A24), we get

$$\begin{aligned}\mathbf{S}_{t|t} - \mathbf{S}_{t|t}^* &= (\mathbf{I} - \mathbf{K}_t\tilde{\mathbf{v}}_t')(\mathbf{S}_{t-1|t-1} - \mathbf{S}_{t-1|t-1}^*)(\mathbf{I} - \mathbf{K}_t\tilde{\mathbf{v}}_t')' + (\mathbf{I} - \mathbf{K}_t\tilde{\mathbf{v}}_t')\mathbf{S}(\mathbf{I} - \mathbf{K}_t\tilde{\mathbf{v}}_t')' \\ &= \mathbf{A}_t(\mathbf{S}_{t-1|t-1} - \mathbf{S}_{t-1|t-1}^*)\mathbf{A}_t' + \mathbf{A}_t\mathbf{S}\mathbf{A}_t',\end{aligned} \tag{A25}$$

where $\mathbf{A}_t = \mathbf{I} - \mathbf{K}_t\tilde{\mathbf{v}}_t' = \mathbf{S}_{t|t}\mathbf{S}_{t|t-1}^{-1}$. Equation (A25) is similar to the discrete Lyapunov equation [11]. Since $\mathbf{A}_t$ is stable, which is proved in [9], the limit of $\mathbf{S}_{t|t} - \mathbf{S}_{t|t}^*$ can be written as

$$\lim_{t\to\infty} \mathbf{S}_{t|t} - \mathbf{S}_{t|t}^* = \sum_{t=1}^{\infty} (\mathbf{A}_t \times \cdots \times \mathbf{A}_1)\mathbf{S}(\mathbf{A}_1' \times \cdots \times \mathbf{A}_t'). \tag{A26}$$

(A26) is hard to compute since $\mathbf{A}_t = \mathbf{S}_{t|t}\mathbf{S}_{t|t-1}^{-1}$ is periodic at steady state because $\mathbf{S}_{t|t}$ and $\mathbf{S}_{t|t-1}$ converge to periodic solutions, respectively. Here we do another approximation—replacing $\mathbf{S}_{t|t}$ and $\mathbf{S}_{t|t-1}$ with their average values at steady state $\widetilde{\mathbf{S}}_+$ and $\widetilde{\mathbf{S}}_- = \widetilde{\mathbf{S}}_+ + \mathbf{S}$, respectively. So $\mathbf{A}_t \approx \widetilde{\mathbf{S}}_+(\widetilde{\mathbf{S}}_+ + \mathbf{S})^{-1}$ and substituting this into (A26), we have

$$\begin{aligned}\widetilde{\mathbf{S}}_+ - \mathbf{S}_+^* &= \sum_{m=1}^{\infty} [\widetilde{\mathbf{S}}_+(\widetilde{\mathbf{S}}_+ + \mathbf{S})^{-1}]^m \mathbf{S}[(\widetilde{\mathbf{S}}_+ + \mathbf{S})^{-1\prime}\widetilde{\mathbf{S}}_+']^m \\ &= s\sum_{m=1}^{\infty} [\widetilde{\mathbf{S}}_+(\widetilde{\mathbf{S}}_+ + \mathbf{S})^{-1}]^m [(\widetilde{\mathbf{S}}_+ + \mathbf{S})^{-1}\widetilde{\mathbf{S}}_+]^m.\end{aligned} \tag{A27}$$

Considering that $\widetilde{\mathbf{S}}_+$ is an analytic function of the learning rate s in (A21) and $\mathbf{S} = s\mathbf{I}$, (A27) shows that $\mathbf{S}_+^*$ can be expressed as an analytic function of s. To write this explicitly, we use the Eigenvalue decomposition of $\widetilde{\mathbf{S}}_+$. Remember that $\widetilde{\mathbf{S}}_+ = \mathbf{U}\ diag(\kappa_1, ..., \kappa_n)\ \mathbf{U}'$, where $\mathbf{U}$ is a unitary matrix. Since $\mathbf{S}$ is diagonal, we have that

$$\widetilde{\mathbf{S}}_+(\widetilde{\mathbf{S}}_+ + \mathbf{S})^{-1} = \mathbf{U}\ diag(\frac{\kappa_1}{\kappa_1 + s}, ..., \frac{\kappa_n}{\kappa_n + s})\ \mathbf{U}'. \tag{A28}$$

Since the above matrix is symmetric, we also have that $[\widetilde{\mathbf{S}}_+(\widetilde{\mathbf{S}}_+ + \mathbf{S})^{-1}] = [\widetilde{\mathbf{S}}_+(\widetilde{\mathbf{S}}_+ + \mathbf{S})^{-1}]' = [(\widetilde{\mathbf{S}}_+ + \mathbf{S})^{-1}\widetilde{\mathbf{S}}_+]$. Taken together equation (A27) can be written as

$$\begin{aligned}
\widetilde{\mathbf{S}}_+ - \mathbf{S}_+^* &= s\sum_{m=1}^{\infty}[\mathbf{U}\ diag(\frac{\kappa_1}{\kappa_1 + s}, ..., \frac{\kappa_n}{\kappa_n + s})\ \mathbf{U}']^{2m} \\
&= \mathbf{U}\ (s\sum_{m=1}^{\infty}\begin{bmatrix}(\frac{\kappa_1}{\kappa_1+s})^{2m} & & \\ & \ddots & \\ & & (\frac{\kappa_n}{\kappa_n+s})^{2m}\end{bmatrix})\ \mathbf{U}' \\
&= \mathbf{U}\begin{bmatrix}\frac{\kappa_1^2}{2\kappa_1+s} & & \\ & \ddots & \\ & & \frac{\kappa_n^2}{2\kappa_n+s}\end{bmatrix}\mathbf{U}',
\end{aligned} \tag{A29}$$

where the last line follows from writing the expression for a geometric sum of series and simplifying. Finally, we can approximate the steady-state error covariance $\mathbf{S}_+^*$ as

$$\mathbf{S}_+^* = \mathbf{U}\begin{bmatrix}\frac{\kappa_1^2+s\kappa_1}{2\kappa_1+s} & & \\ & \ddots & \\ & & \frac{\kappa_n^2+s\kappa_n}{2\kappa_n+s}\end{bmatrix}\mathbf{U}'.$$

This is equation (7) in theorem 1. Now we have an analytical expression for the steady-state error covariance as a function of the learning rate s. In the next section we derive the convergence rate as a function of s, which is the second result, equation (8), in theorem 1.

Analytical Function for the Convergence Time

Ensuring that the convergence time of error $\mathrm{E}[\mathbf{g}_t]$ is below an upper-bound value—while minimizing the steady-state error covariance—is another objective for which we can design the calibration algorithm to select the learning rate s. To derive the calibration algorithm corresponding to this objective, here we find an analytic function that relates the convergence time to the learning rate. We again start from the error dynamics equation in (A18). Taking expectation over both sides of (A18), we have that

$$\begin{aligned}
\mathrm{E}[\mathbf{g}_t] &= (\mathbf{I} - \mathbf{K}_t\hat{\mathbf{v}}_t')\mathrm{E}[\mathbf{g}_{t-1}] \\
&= (\mathbf{S}_{t|t}\mathbf{S}_{t|t-1}^{-1})\mathrm{E}[\mathbf{g}_{t-1}].
\end{aligned} \tag{A30}$$

From (A30), the convergence time is determined by $\mathbf{S}_{t|t}$ and $\mathbf{S}_{t|t-1}$ during the transient state of the KF and before steady state; however, the transient values of the covariances are dependent on the initialization in the KF and are not computable in general. Hence to enable the computation of the convergence time, we set the initial value of the parameter covariance in the KF as $\mathbf{S}_{0|0} = \widetilde{\mathbf{S}}_+$ so that the covariance matrix is already at steady state even initially. This is practical since the initialization $\mathbf{S}_{0|0}$ is a user's choice and since $\widetilde{\mathbf{S}}_+$ can be calculated offline from (A19) and (A20). Both (A19) and (A20) are independent of neural observations $\{y_t\}$. Thus we can derive the convergence time under this initialization choice.

Since $\mathbf{S}_{0|0} = \widetilde{\mathbf{S}}_{+}$, we could approximate $\mathbf{S}_{t|t}$ and $\mathbf{S}_{t|t-1}$ as their steady-state values, $\widetilde{\mathbf{S}}_{+}$ and $\widetilde{\mathbf{S}}_{-} = \widetilde{\mathbf{S}}_{+} + \mathbf{S}$ in (A30), respectively. So using (A28), (A30) becomes

$$\begin{aligned} \mathrm{E}[\mathbf{g}_t] &= \widetilde{\mathbf{S}}_{+}(\widetilde{\mathbf{S}}_{+} + \mathbf{S})^{-1} \times \mathrm{E}[\mathbf{g}_{t-1}] \\ &= (\mathbf{U} \begin{bmatrix} \frac{\kappa_1}{\kappa_1 + s} & & \\ & \ddots & \\ & & \frac{\kappa_n}{\kappa_n + s} \end{bmatrix} \mathbf{U}') \times \mathrm{E}[\mathbf{g}_{t-1}]. \end{aligned}$$

This is equation (8) in theorem 1.

Appendix E: Generalizing the Calibration Algorithm to Non-Periodic Training States

Here we show why we assumed periodicity of the behavioral state $\tilde{\mathbf{v}}_t$ during training in our mathematical derivation of the calibration algorithm, and also demonstrate what extra assumption we need in order to extend the derivation to the non-periodic case. As shown in Figs 5 and 6B, the calibration algorithm is also accurate in the non-periodic case; as we will show below, this is because this extra assumption is relatively mild. For conciseness, we use the KF case as an example. S2 Fig shows the sketch of the derivations for periodic and non-periodic cases to show that their distinction is in this one assumption.

Appendix D shows that to derive the analytic calibration algorithm, we need to analytically compute the average steady-state value of the prediction covariance $\mathbf{S}_{t+1|t}$, which is the solution of a time-varying DRE equation (equation (A17) in Appendix C). However, before we do so, the first step is to ensure that this average exists theoretically. It is precisely for ensuring this existence that we required the periodicity of the behavioral state $\tilde{\mathbf{v}}_t$ in the training session. This is because here the DRE coefficients are a function of $\tilde{\mathbf{v}}_t$ (equation (A17) in Appendix C); moreover, as shown in Theorem 6.12 in reference [9], when the coefficients of the DRE are periodic, the solution $\mathbf{S}_{t+1|t}$ of the corresponding discrete periodic Riccati equation (DPRE) would converge toward a symmetric, periodic and positive definite (SPPD) solution. Consequently, since in this case $\mathbf{S}_{t+1|t}$ would become periodic, it would have a bounded and theoretically well-defined average.

In contrast, if the coefficient $\tilde{\mathbf{v}}_t$ of the DRE is not periodic but random, we don't know if $\mathbf{S}_{t+1|t}$ has bounded steady-state moments or not, so we cannot guarantee that its steady-state average exists. That is why for rigorous derivation of the calibration algorithm, we considered periodic $\tilde{\mathbf{v}}_t$ in the training session. So the only reason for assuming periodicity is to ensure the existence of the average of $\mathbf{S}_{t+1|t}$ at steady state. After this step, we don't need the periodicity assumption. That is precisely why the calibration algorithm still applies to the non-periodic case if we have a well-behaved $\mathbf{S}_{t+1|t}$ with a bounded steady-state average, which is a relatively mild requirement. The periodicity assumption simply guarantees the existence of the average of $\mathbf{S}_{t+1|t}$ at steady state for derivation purposes, instead of assuming this existence.

Once we know the steady-state average of $\mathbf{S}_{t+1|t}$ exists (whether in the periodic or non-periodic case), we can compute it analytically through a second step. In this second step, since the DRE (equation (A17) in Appendix C) in the calibration algorithm has time-variant coefficients, we first approximate this time-variant DRE with a time-invariant one by computing the expected value of the coefficients from the training data. We then derive the solution for this special DRE with time-invariant (i.e., constant) coefficients in Appendix J to finally compute the steady-state average of $\mathbf{S}_{t+1|t}$. We show in Appendix D that, if the average exists, we can estimate it by calculating $\mathbf{H}_{ave}$, which is the average of $\tilde{\mathbf{v}}_t\tilde{\mathbf{v}}_t'Z^{-1}$. So for the periodic case we compute this average over one period and for the general non-periodic case (if we just assume that $\mathbf{S}_{t+1|t}$ has bounded steady-state moments), we can take the average over all time (i.e., set $T \to \infty$). Indeed our numerical simulations demonstrate that this averaging works for random non-periodic coefficients (Figs 5 and 6B).

In summary, the reason that we keep periodicity throughout the derivation of the calibration algorithm is for making it more mathematically rigorous. But in practice, the same algorithm can be applied on non-periodic cases. Intuitively, even for a very large period, periodicity of a variable still ensures that the variable needs to come back to its initial value at some point, and will therefore have a well-defined bounded average. If we don't assume periodicity (i.e., infinite period), the variable does not have to come back to its initial value and thus, for example, could even be unbounded with an unbounded average. Thus we cannot ensure that the average exists. Again as we indicated above, we can simply assume that the average exists—which is a relatively mild assumption—and extend the derivation to the non-periodic case (see S2 Fig).

Appendix F: The Inverse Functions for the Convergence Time and the Steady-State Error Covariance in KF

Here we show how we derive (9) and (10) from (7) and (8).

Learning rate analytical expression for a given steady-state error covariance. We first derive the inverse function to compute the learning rate for a given steady-state error covariance. The goal is to solve for the learning rate s from the inequality $\lim_{t\to\infty} \|\mathrm{Cov}[\boldsymbol{\psi}_{t|t}]\| \le V_{bd}$. Note that $\lim_{t\to\infty} \|\mathrm{Cov}[\boldsymbol{\psi}_{t|t}]\| = \|\mathbf{S}_+^*\|$ is the largest eigenvalue of $\mathbf{S}_+^*$ due to its positive definite property. From theorem 1, each eigenvalue of $\mathbf{S}_+^*$ can be expressed as

$$\frac{\kappa_m^2 + s\kappa_m}{2\kappa_m + s} = \frac{s}{\sqrt{h_m^2 s^2 + 4h_m s}} \le V_{bd} \quad (m = 1, ..., n). \tag{A31}$$

The maximal eigenvalue corresponds to the minimal h_m, which is h_1. After some algebraic manipulations, this optimal learning rate is given by

$$s = \frac{4h_1}{\frac{1}{V_{bd}^2} - h_1^2} \quad \text{with} \quad \frac{1}{V_{bd}^2} > h_1^2.$$

This is equation (9) in theorem 2.

Learning rate analytical expression for a given convergence time. We now find the inverse function for calculating the learning rate from the convergence time constraint. By taking norm on both sides of (8), we have

$$\|\mathrm{E}[\mathbf{g}_t]\| \le \frac{\kappa_1}{\kappa_1 + s} \times \|\mathrm{E}[\mathbf{g}_{t-1}]\|.$$

Remember that the goal is calculating the learning rate s that could make $\frac{\|\mathrm{E}[\mathbf{g}_t]\|}{\|\mathrm{E}[\mathbf{g}_0]\|} \le E_{rest}$ before the time given by the upper-bound of the convergence time C_{bd}. Assuming each step taking Δ seconds, there are approximately $\frac{C_{bd}}{\Delta}$ steps before the given time constraint C_{bd}. Combining it with the above equation, we can write the mathematical expression of this optimization problem as

$$\|\mathrm{E}[\mathbf{g}_{\frac{C_{bd}}{\Delta}}]\| \le (\frac{\kappa_1}{\kappa_1 + s})^{\frac{C_{bd}}{\Delta}} \times \|\mathrm{E}[\mathbf{g}_0]\| \le E_{rest} \times \|\mathrm{E}[\mathbf{g}_0]\|.$$

Now since from (A21)

$$(\frac{\kappa_1}{\kappa_1 + s})^{\frac{C_{bd}}{\Delta}} = [\frac{s}{h_1} \times \frac{4h_1^2}{(\sqrt{h_1^2 s^2 + 4h_1 s} + h_1 s)^2}]^{\frac{C_{bd}}{\Delta}},$$

the last inequality is equivalent to

$$[\frac{s}{h_1} \times \frac{4h_1^2}{(\sqrt{h_1^2 s^2 + 4h_1 s} + h_1 s)^2}]^{\frac{C_{bd}}{\Delta}} \le E_{rest}.$$

This equation can be simplified as

$$\frac{s}{(\sqrt{h_1^2 s^2 + 4h_1 s} + h_1 s)^2} \le \frac{1}{4h_1} \times (E_{rest})^{\frac{\Delta}{C_{bd}}}.$$

Defining $C_{time} = \frac{1}{4h_1} \times (E_{rest})^{\frac{\Delta}{C_{bd}}}$, which is independent from the learning rate s, and after some algebraic manipulations, the optimal learning rate is given by

$$s = \frac{C_{time}}{4h_1^2} \times (\frac{1}{C_{time}} - 4h_1)^2.$$

This is equation (10) in theorem 2.

Appendix G: PPF is Asymptotically Unbiased

Here we show that the posterior mean $\boldsymbol{\phi}_{t|t}$ in (18) is asymptotically unbiased and the posterior covariance $\mathbf{Q}_{t|t}$ converges to a symmetric, periodic, and positive definite (SPPD) solution in PPF. We want to find a recursion for the expected value $\mathrm{E}[\boldsymbol{\phi}_{t|t}]$ using the nonlinear equations in (15)–(18). Given the nonlinearity of (15)–(18), we perform an approximation by replacing the random variable, N_t, in (18) with its expected value, $\lambda(t|\boldsymbol{\phi}^*)\Delta = \mathrm{E}[N_t]$. This is also the instantaneous firing rate. Since the only random variable N_t in (15)–(18) is replaced by its expected value, the series $\{\boldsymbol{\phi}_{t|t}\}$ becomes deterministic in this case. Let's denote this deterministic series by $\{\tilde{\boldsymbol{\phi}}_{t|t}\}$ to distinguish it from the original stochastic series $\{\boldsymbol{\phi}_{t|t}\}$. Thus the expected value of the original series $\mathrm{E}[\boldsymbol{\phi}_{t|t}]$ can be approximated by $\{\tilde{\boldsymbol{\phi}}_{t|t}\}$. Intuitively, $\tilde{\boldsymbol{\phi}}_{t|t}$ is the best value for describing the instantaneous firing rates from time 1 to t—and consequently the neural spike observations $N_{1:t}$—with the corresponding $\{\tilde{\mathbf{v}}_{1:t}\}$. Since all firing rates have the same constant parameter $\boldsymbol{\phi}$, intuitively, there should be only one optimal value for describing all firing rates and consequently all neural spike observations. This implies that the limit of $\{\tilde{\boldsymbol{\phi}}_{t|t}\}$, which is estimated using these observations, should exist. The existence of this limit is verified by our numerical simulations in the Results section (Fig 7). We now show that if the limit of $\{\tilde{\boldsymbol{\phi}}_{t|t}\}$ exists, then $\lim_{t\to\infty} \mathrm{E}[\boldsymbol{\phi}_{t|t}] \approx \lim_{t\to\infty} \tilde{\boldsymbol{\phi}}_{t|t} = \boldsymbol{\phi}^*$. This would thus imply that the original series $\{\boldsymbol{\phi}_{t|t}\}$ in (15)–(18) is unbiased.

Lemma 1. *Let's denote by* $\{\tilde{\boldsymbol{\phi}}_{t|t}\}$ *the series in* (15)*–*(18) *that is obtained by replacing* N_t *with* $\lambda(t|\boldsymbol{\phi}^*)\Delta$ *in* (18)*. Let's assume that* $\lim_{t\to\infty} \tilde{\boldsymbol{\phi}}_{t|t}$ *exists. Then* $\lim_{t\to\infty} \tilde{\boldsymbol{\phi}}_{t|t} = \boldsymbol{\phi}^*$ *(i.e., the solution is unique.)*

Proof. Denote $\lim_{t\to\infty} \tilde{\boldsymbol{\phi}}_{t|t} = \widehat{\boldsymbol{\phi}}$. Taking $t \to \infty$ in (18), it becomes

$$\widehat{\boldsymbol{\phi}} = \widehat{\boldsymbol{\phi}} + \lim_{t\to\infty} \mathbf{Q}_{t|t}\tilde{\mathbf{v}}_t[\lambda(t|\boldsymbol{\phi}^*) - \lambda(t|\widehat{\boldsymbol{\phi}})]\Delta. \tag{A32}$$

Since $\mathbf{Q}_{t|t}$ is nonsingular and $\tilde{\mathbf{v}}_t$ explores the dynamic range of possible behavioral states in a well-designed training experiment (i.e., it is not zero most of the time), for (A32) to hold, we must have $\lambda(t|\boldsymbol{\phi}^*) - \lambda(t|\widehat{\boldsymbol{\phi}}) = 0$ for $\forall t$. Remember that $\lambda(t|\cdot)$ is log-linear. We thus can write the difference as

$$\begin{aligned}\lambda(t|\boldsymbol{\phi}^*) - \lambda(t|\widehat{\boldsymbol{\phi}}) &= \exp(\tilde{\mathbf{v}}_t'\boldsymbol{\phi}^*) - \exp(\tilde{\mathbf{v}}_t'\widehat{\boldsymbol{\phi}}) \\ &= \exp(\tilde{\mathbf{v}}_t'\boldsymbol{\phi}^*) \times [1 - \exp(\tilde{\mathbf{v}}_t'(\widehat{\boldsymbol{\phi}} - \boldsymbol{\phi}^*))] \\ &= 0 \text{ for } \forall t \geq 0. \end{aligned} \tag{A33}$$

So for the above relation to hold we must have $1 = \exp(\tilde{\mathbf{v}}_t'(\widehat{\boldsymbol{\phi}} - \boldsymbol{\phi}^*))$, which implies that $\tilde{\mathbf{v}}_t'(\widehat{\boldsymbol{\phi}} - \boldsymbol{\phi}^*) = 0$ for all t. Thus $\widehat{\boldsymbol{\phi}} = \boldsymbol{\phi}^*$. □

Note that in the proof of lemma 1, we assume that $\lim_{t\to\infty} \mathbf{Q}_{t|t}$ is nonsingular. As noted above, in a well-designed training experiment this will be the case as also confirmed in our numerical simulations in the Results section. Further, this nonsingularity can be inferred by observing that $\{\mathbf{Q}_{t|t-1}\}$ will converge to a SPPD solution as follows. Due to the similarity between equation sets (4)–(5) and (16)–(17), we can derive the recursive equation for $\mathbf{Q}_{t|t-1}$ by following the same steps as in Appendix C. This recursive equation is

$$\mathbf{Q}_{t+1|t} = \mathbf{Q}_{t|t-1} + \mathbf{Q} - \frac{\mathbf{Q}_{t|t-1}\tilde{\mathbf{v}}_t\tilde{\mathbf{v}}_t'\mathbf{Q}_{t|t-1}}{k_t}, \tag{A34}$$

where $k_t = (\lambda(t|\boldsymbol{\phi}_{t|t-1})\Delta)^{-1} + \tilde{\mathbf{v}}_t'\mathbf{Q}_{t|t-1}\tilde{\mathbf{v}}_t$ is a time dependent scalar. If the coefficients (i.e., $\tilde{\mathbf{v}}_t$ and $\lambda(t|\boldsymbol{\phi}_{t|t-1})$) are periodic (which is approximately the case for us), (A34) becomes DPRE and $\{\mathbf{Q}_{t|t-1}\}$ will converge to a SPPD solution from Appendix C. Numerical simulation again confirmed this convergence to an (approximate) SPPD solution. Note that $\mathbf{Q}_{t|t}$ also converges to a SPPD solution from (16). So $\mathbf{Q}_{t|t-1}$ and $\mathbf{Q}_{t|t}$ are nonsingular. These results are also useful in deriving the calibration algorithm for PPF in Appendix H.

Appendix H: Derivation of the Steady-State Error Covariance as a Function of the Learning Rate r in PPF

Here we derive equation (19), an analytic expression of the steady-state error covariance $\mathbf{Q}_+^*$ with respect to the learning rate r, in theorem 3. Our derivation will be similar to that for the continuous neural signals and KF in Appendix D. We first express the estimation covariance $\mathbf{Q}_{t|t}$ in (17) and the difference $\mathbf{Q}_{t|t} - \mathbf{Q}_{t|t}^*$ (where $\mathbf{Q}_{t|t}^*$ is the true parameter error covariance) as functions of the learning rate r. We then take the limit $t \to \infty$ to write the steady-state error covariance $\mathbf{Q}_+^* = \lim_{t\to\infty} \mathbf{Q}_{t|t}^*$ as an analytic function of r.

From (15) and (18), the dynamics model of the parameter error $\mathbf{e}_t = \boldsymbol{\phi}^* - \boldsymbol{\phi}_{t|t}$ is

$$\mathbf{e}_t = \mathbf{e}_{t-1} - \mathbf{Q}_{t|t}\tilde{\mathbf{v}}_t[N_t - \lambda(t|\boldsymbol{\phi}_{t-1|t-1})]\Delta. \quad \text{(A35)}$$

As in the case of the continuous signal, the average values of $\mathbf{Q}_{t|t}$ and $\mathbf{Q}_{t|t-1}$ are critical in deriving $\mathbf{Q}_+^*$ from (A35). Next, we first find $\mathbf{Q}_{t|t}$ and $\mathbf{Q}_{t|t-1}$ at steady state and then solve for the steady-state error covariance $\mathbf{Q}_+^*$ from (A35).

Analytical Function for the Steady-State Error Covariance

Here, we first find the steady-state average of $\mathbf{Q}_{t|t}$, denoted by $\widetilde{\mathbf{Q}}_+$, as an analytic function of the learning rate. We then write the steady-state error covariance $\mathbf{Q}_+^* = \lim_{t\to\infty} \mathbf{Q}_{t|t}^*$ in terms of this average and hence in terms of the learning rate. As in theorem 3, we assume that the state, e.g., the intended velocity $\{\mathbf{v}_t\}$, is periodic with period T for rigorousness in derivation to ensure that the steady-state average values exist theoretically. From Appendix G, $\mathbf{Q}_{t|t}$ and $\mathbf{Q}_{t|t-1}$ in (16) and (17) will converge to SPPD solutions. We denote the average of $\mathbf{Q}_{t|t}$ and that of $\mathbf{Q}_{t|t-1}$ at steady state by $\widetilde{\mathbf{Q}}_+$ and $\widetilde{\mathbf{Q}}_-$, respectively. Remember that the noise covariance matrix is $\mathbf{Q} = r\mathbf{I}_n$ $(r > 0)$ and r is the learning rate. As before, we take an average over both sides of (16) and (17) to get

$$\widetilde{\mathbf{Q}}_- = \widetilde{\mathbf{Q}}_+ + \mathbf{Q} \quad \text{(A36)}$$

$$\widetilde{\mathbf{Q}}_+^{-1} = \widetilde{\mathbf{Q}}_-^{-1} + \frac{1}{T}\sum_{t=1}^{T} \tilde{\mathbf{v}}_t\tilde{\mathbf{v}}_t'\lambda(t|\boldsymbol{\phi}^*)\Delta \quad \text{(A37)}$$

Note that we replace $\lambda(t|\boldsymbol{\phi}_{t|t-1})$ with $\lambda(t|\boldsymbol{\phi}^*)$ in (A37) since at steady state, $\mathrm{E}[\boldsymbol{\phi}_{t|t-1}] = \mathrm{E}[\boldsymbol{\phi}_{t-1|t-1}] \approx \boldsymbol{\phi}^*$ from Appendix G. We define $\mathbf{M}_{ave} = \frac{1}{T}\sum_{t=1}^{T} \tilde{\mathbf{v}}_t\tilde{\mathbf{v}}_t'\lambda(t|\boldsymbol{\phi}^*)\Delta$. As we derive in Appendix J, we can express $\widetilde{\mathbf{Q}}_+$ as an analytic function of the learning rate r as follows. Denoting the eigenvalue decomposition of $\mathbf{M}_{ave} = \mathbf{U}\ diag(a_1, ..., a_n)\ \mathbf{U}'$ $(a_i \leq a_{i+1})$, we have $\widetilde{\mathbf{Q}}_+ = \mathbf{U}\ diag(b_1, ..., b_n)\ \mathbf{U}'$ with eigenvalues

$$b_m = \frac{\sqrt{a_m^2 r^2 + 4a_m r} - a_m r}{2a_m} \quad \text{(A38)}$$

$$a_m = \frac{1}{b_m} - \frac{1}{b_m + r}. \quad \text{(A39)}$$

So we now can express $\widetilde{\mathbf{Q}}_+$, the steady-state average of $\mathbf{Q}_{t|t}$, as an analytic function of the learning rate r given above. Hence if we can also write $\mathbf{Q}_{t|t} - \mathbf{Q}_{t|t}^*$ as an analytic function, then we know the explicit connection between the steady-state error covariance $\mathbf{Q}_+^* = \lim_{t\to\infty} \mathbf{Q}_{t|t}^*$ and the learning rate r. Below, we derive $\mathbf{Q}_{t|t} - \mathbf{Q}_{t|t}^*$ from a recursive equation for the error.

To solve for the steady-state error covariance $\mathbf{Q}_+^*$ from (A35), we first derive a recursive equation for $\mathbf{Q}_{t|t}^*$ and then take the limit. To do this, we first write a recursive equation for the error $\mathbf{e}_t$. We can write the last term of (A35) as

$$\mathbf{e}_t = \mathbf{e}_{t-1} - \mathbf{Q}_{t|t}\tilde{\mathbf{v}}_t[\lambda(t|\boldsymbol{\phi}^*) - \lambda(t|\boldsymbol{\phi}_{t-1|t-1})]\Delta - \mathbf{Q}_{t|t}\tilde{\mathbf{v}}_t[N_t - \lambda(t|\boldsymbol{\phi}^*)\Delta]. \quad \text{(A40)}$$

Since at steady state, $\mathrm{E}[\mathbf{e}_t] \to \mathbf{0}$ from Appendix G, we approximate $\lambda(t|\boldsymbol{\phi}^*) - \lambda(t|\boldsymbol{\phi}_{t-1|t-1})$ using its Taylor series expansion as

$$\begin{aligned}\lambda(t|\boldsymbol{\phi}^*) - \lambda(t|\boldsymbol{\phi}_{t-1|t-1}) &= \exp(\tilde{\mathbf{v}}_t'\boldsymbol{\phi}^*) - \exp(\tilde{\mathbf{v}}_t'\boldsymbol{\phi}_{t-1|t-1}) \\ &= \exp(\tilde{\mathbf{v}}_t'\boldsymbol{\phi}_{t-1|t-1}) \times [\exp(\tilde{\mathbf{v}}_t'(\boldsymbol{\phi}^* - \boldsymbol{\phi}_{t-1|t-1})) - 1] \\ &= \exp(\tilde{\mathbf{v}}_t'\boldsymbol{\phi}_{t-1|t-1}) \times [\exp(\tilde{\mathbf{v}}_t'\mathbf{e}_{t-1}) - 1] \\ &\approx \exp(\tilde{\mathbf{v}}_t'\boldsymbol{\phi}_{t-1|t-1}) \times (\tilde{\mathbf{v}}_t'\mathbf{e}_{t-1}) \\ &= \lambda(t|\boldsymbol{\phi}_{t-1|t-1}) \times (\tilde{\mathbf{v}}_t'\mathbf{e}_{t-1}). \end{aligned} \tag{A41}$$

The third line gives the fourth line using the Taylor series expansion of the exponential function and because of the closeness of $\mathbf{e}_{t-1}$ and $\mathbf{0}$ at steady state. Substituting (A41) in (A40), (A40) becomes

$$\begin{aligned}\mathbf{e}_t &= \mathbf{e}_{t-1} - \mathbf{Q}_{t|t}\tilde{\mathbf{v}}_t\tilde{\mathbf{v}}_t'\mathbf{e}_{t-1}\lambda(t|\boldsymbol{\phi}_{t-1|t-1})\Delta - \mathbf{Q}_{t|t}\tilde{\mathbf{v}}_t(N_t - \lambda(t|\boldsymbol{\phi}^*)\Delta) \\ &= [\mathbf{I} - \mathbf{Q}_{t|t}\tilde{\mathbf{v}}_t\tilde{\mathbf{v}}_t'\lambda(t|\boldsymbol{\phi}_{t-1|t-1})\Delta]\,\mathbf{e}_{t-1} - \mathbf{Q}_{t|t}\tilde{\mathbf{v}}_t(N_t - \lambda(t|\boldsymbol{\phi}^*)\Delta) \\ &= \mathbf{Q}_{t|t}\mathbf{Q}_{t|t-1}^{-1}\mathbf{e}_{t-1} - \mathbf{Q}_{t|t}\tilde{\mathbf{v}}_t(N_t - \lambda(t|\boldsymbol{\phi}^*)\Delta), \end{aligned} \tag{A42}$$

where we get the third line from the second line by using (17). This gives a recursive equation for $\mathbf{e}_t$. By defining $\mathbf{F}_t = \mathbf{Q}_{t|t}\mathbf{Q}_{t|t-1}^{-1}$ and taking the covariance of both sides of (A42), the recursive equation for the error covariance $\mathbf{Q}_{t|t}^*$ is given by

$$\mathbf{Q}_{t|t}^* = \mathbf{F}_t\mathbf{Q}_{t-1|t-1}^*\mathbf{F}_t' + \mathbf{Q}_{t|t}\tilde{\mathbf{v}}_t\tilde{\mathbf{v}}_t'\mathbf{Q}_{t|t}[\lambda(t|\boldsymbol{\phi}^*)\Delta(1 - \lambda(t|\boldsymbol{\phi}^*)\Delta)], \tag{A43}$$

where to get the second term we used the fact that N_t is Bernoulli-distributed with parameter $\lambda(t|\boldsymbol{\phi}^*)\Delta$. To calculate $\mathbf{Q}_{t|t} - \mathbf{Q}_{t|t}^*$ for finding the steady-state error covariance $\mathbf{Q}_+^*$, we need the recursive equation of $\mathbf{Q}_{t|t}$. From (16) and (17), this recursive function can be written in the Joseph form of the covariance matrix [10] as follows

$$\mathbf{Q}_{t|t} = \mathbf{F}_t\mathbf{Q}_{t-1|t-1}\mathbf{F}_t' + \mathbf{F}_t\mathbf{Q}\mathbf{F}_t' + \mathbf{Q}_{t|t}\tilde{\mathbf{v}}_t\tilde{\mathbf{v}}_t'\mathbf{Q}_{t|t}\lambda(t|\boldsymbol{\phi}_{t|t-1})\Delta. \tag{A44}$$

As mentioned above, since $\widetilde{\mathbf{Q}}_+$, the steady-state average of $\mathbf{Q}_{t|t}$, can be expressed as a function of the learning rate r, if we can calculate the difference between $\widetilde{\mathbf{Q}}_+$ and $\mathbf{Q}_{t|t}^*$, we can express the steady-state error covariance $\mathbf{Q}_+^* = \lim_{t\to\infty}\mathbf{Q}_{t|t}^*$ as an analytic function of the learning rate r as well. So we now subtract (A43) from (A44) and approximate $\boldsymbol{\phi}_{t|t-1}$ in (A44) with $\boldsymbol{\phi}^*$. This approximation is reasonable since $\mathrm{E}[\mathbf{e}_{t-1}] \approx \mathbf{0}$ at the steady state from Appendix G. The subtraction gives

$$\mathbf{Q}_{t|t} - \mathbf{Q}_{t|t}^* = \mathbf{F}_t\left[\mathbf{Q}_{t-1|t-1} - \mathbf{Q}_{t-1|t-1}^*\right]\mathbf{F}_t' + \mathbf{F}_t\mathbf{Q}\mathbf{F}_t' + \mathbf{Q}_{t|t}\tilde{\mathbf{v}}_t\tilde{\mathbf{v}}_t'\mathbf{Q}_{t|t}\lambda^2(t|\boldsymbol{\phi}^*)\Delta^2. \tag{A45}$$

Note that $\tilde{\mathbf{v}}_t\tilde{\mathbf{v}}_t'\lambda^2(t|\boldsymbol{\phi}^*)$ is periodic since $\{\mathbf{v}_t\}$ is periodic. (A45) is a discrete Lyapunov equation [11] for $(\mathbf{Q}_{t|t} - \mathbf{Q}_{t|t}^*)$ with time-variant coefficient matrices. From Appendix G, $\mathbf{Q}_{t|t}$ and $\mathbf{Q}_{t|t-1}$ converge to SPPD solutions and since $\mathbf{F}_t = \mathbf{Q}_{t|t}\mathbf{Q}_{t|t-1}^{-1}$, both $\mathbf{Q}_{t|t}$ and $\mathbf{F}_t$ are periodic and bounded. Given these periodic and bounded properties and for computational tractability, we approximate $\mathbf{F}_t$, $\mathbf{Q}_{t|t}$, and $\tilde{\mathbf{v}}_t\tilde{\mathbf{v}}_t'\lambda^2(t|\boldsymbol{\phi}^*)\Delta^2$ with their average values. Note that the average value of $\mathbf{F}_t$ is $\mathbf{F} = \widetilde{\mathbf{Q}}_+(\widetilde{\mathbf{Q}}_+ + \mathbf{Q})^{-1}$ using its definition and (16). The average values of $\mathbf{Q}_{t|t}$, and $\tilde{\mathbf{v}}_t\tilde{\mathbf{v}}_t'\lambda^2(t|\boldsymbol{\phi}^*)\Delta^2$ are $\widetilde{\mathbf{Q}}_+$, and $\frac{1}{T}\sum_{t=1}^{T}\tilde{\mathbf{v}}_t\tilde{\mathbf{v}}_t'\lambda^2(t|\boldsymbol{\phi}^*)\Delta^2$, respectively. Thus (A45) becomes

$$\widetilde{\mathbf{Q}}_+ - \mathbf{Q}_{t|t}^* \approx \mathbf{F}[\widetilde{\mathbf{Q}}_+ - \mathbf{Q}_{t-1|t-1}^*]\mathbf{F}' + \mathbf{F}\mathbf{Q}\mathbf{F}' + \widetilde{\mathbf{Q}}_+\Big(\frac{1}{T}\sum_{t=1}^{T}\tilde{\mathbf{v}}_t\tilde{\mathbf{v}}_t'\lambda^2(t|\boldsymbol{\phi}^*)\Delta^2\Big)\widetilde{\mathbf{Q}}_+. \tag{A46}$$

Note that $\lim_{t\to\infty}\mathbf{Q}_{t|t}^* = \lim_{t\to\infty}\mathbf{Q}_{t-1|t-1}^* = \mathbf{Q}_+^*$, where $\mathbf{Q}_+^*$ is again the true steady-state parameter error covariance. We can now take the limit of the above equation and use the solution of the

discrete Lyapunov equation [11] to get

$$\lim_{t\to\infty}\big(\widetilde{\mathbf{Q}}_{+}-\mathbf{Q}^{*}_{t|t}\big)=\widetilde{\mathbf{Q}}_{+}-\mathbf{Q}^{*}_{+}$$
$$=\sum_{k=0}^{\infty}\mathbf{F}^{k}(\mathbf{F}\mathbf{Q}\mathbf{F}'+\widetilde{\mathbf{Q}}_{+}\Big(\frac{1}{T}\sum_{t=1}^{T}\tilde{\mathbf{v}}_t\tilde{\mathbf{v}}_t'\lambda^2(t|\boldsymbol{\phi}^*)\Delta^2\Big)\widetilde{\mathbf{Q}}_{+})(\mathbf{F}')^{k}. \tag{A47}$$

For conciseness, define $\boldsymbol{\Omega}_{ave}=\sum_{k=0}^{\infty}\mathbf{F}^{k}\widetilde{\mathbf{Q}}_{+}\big(\frac{1}{T}\sum_{t=1}^{T}\tilde{\mathbf{v}}_t\tilde{\mathbf{v}}_t'\lambda^2(t|\boldsymbol{\phi}^*)\Delta^2\big)\widetilde{\mathbf{Q}}_{+}(\mathbf{F}')^{k}$. Remember that $\mathbf{F}=\mathbf{F}'$ is a symmetric matrix, $\mathbf{F}=\widetilde{\mathbf{Q}}_{+}(\widetilde{\mathbf{Q}}_{+}+\mathbf{Q})^{-1}=\mathbf{U}\ diag(\frac{b_i}{b_i+r})\ \mathbf{U}'$ with $(i=1,...,n)$, and $\mathbf{Q}=r\mathbf{I}_n$ where r is the learning rate. We now get the steady-state error covariance matrix $\mathbf{Q}^*_{+}$ as

$$\begin{aligned}
\mathbf{Q}^*_{+}&=\widetilde{\mathbf{Q}}_{+}-\sum_{k=0}^{\infty}\mathbf{F}^{k+1}\mathbf{Q}(\mathbf{F}')^{k+1}-\boldsymbol{\Omega}_{ave}\\
&=\widetilde{\mathbf{Q}}_{+}-r\times\sum_{k=0}^{\infty}\mathbf{F}^{2k+2}-\boldsymbol{\Omega}_{ave}\\
&=\widetilde{\mathbf{Q}}_{+}-\mathbf{U}\ (r\sum_{k=0}^{\infty}\begin{bmatrix}(\frac{b_1}{b_1+r})^{2k+2}&&\\&\ddots&\\&&(\frac{b_n}{b_n+r})^{2k+2}\end{bmatrix})\ \mathbf{U}'-\boldsymbol{\Omega}_{ave}\\
&=\mathbf{U}\begin{bmatrix}b_1&&\\&\ddots&\\&&b_n\end{bmatrix}\mathbf{U}'-\mathbf{U}\begin{bmatrix}\frac{b_1^2}{2b_1+r}&&\\&\ddots&\\&&\frac{b_n^2}{2b_n+r}\end{bmatrix}\mathbf{U}'-\boldsymbol{\Omega}_{ave}\\
&=\mathbf{U}\begin{bmatrix}\frac{b_1^2+b_1r}{2b_1+r}&&\\&\ddots&\\&&\frac{b_n^2+b_nr}{2b_n+r}\end{bmatrix}\mathbf{U}'-\boldsymbol{\Omega}_{ave}.
\end{aligned}$$

Note that the third line gives the fourth line by using the well-known formula for the geometric series in the summation and using the identity $\widetilde{\mathbf{Q}}_{+}=\mathbf{U}\ diag(b_1,...,b_n)\ \mathbf{U}'$. If we compare the last line with (7) since both $\mathbf{Q}^*_{+}$ and $\mathbf{S}^*_{+}$ are steady-state error covariances, we see that the last line has an additional term, $\boldsymbol{\Omega}_{ave}$. Practically, this term is very small since (12) is a good approximation of a Bernoulli process and the firing probability, $\lambda(t|\boldsymbol{\phi}^*)\Delta$, is small. Hence we can ignore $\boldsymbol{\Omega}_{ave}$ in calculating $\mathbf{Q}^*_{+}$. Doing so, the steady-state error covariance, $\mathbf{Q}^*_{+}$, for the case of spikes becomes (19) in theorem 3 and it has the same form as the equation obtained for the steady-state error covariance for continuous signals given in (7).

Appendix I: The Approximation in $\widetilde{\mathrm{S}}_+$ Derivation

Here we show that the following holds when the learning rate s is small. This approximation is used in the derivation of the steady-state error covariance.

$$(\frac{1}{T}\sum_{t=t^*+1}^{t^*+T}\mathbf{S}_{t|t})^{-1}-\frac{1}{T}\sum_{t=t^*+1}^{t^*+T}\mathbf{S}_{t|t}^{-1}\approx(\frac{1}{T}\sum_{t=t^*+1}^{t^*+T}\mathbf{S}_{t|t-1})^{-1}-\frac{1}{T}\sum_{t=t^*+1}^{t^*+T}\mathbf{S}_{t|t-1}^{-1}. \tag{A48}$$

To show the above, we first prove the following lemma.

Lemma 2. *Consider* $\mathbf{Y}_n=\mathbf{X}_n+s\mathbf{I}$ *for* $n\in\{1,\cdots,N\}$*, we define the following symbols:*

$$\begin{aligned}\mathbf{V}&=\frac{1}{N}\sum_n\mathbf{X}_n^{-1} \qquad \widehat{\mathbf{V}}=\big(\frac{1}{N}\sum_n\mathbf{X}_n\big)^{-1}\\ \mathbf{W}&=\frac{1}{N}\sum_n\mathbf{Y}_n^{-1} \qquad \widehat{\mathbf{W}}=\big(\frac{1}{N}\sum_n\mathbf{Y}_n\big)^{-1}\end{aligned}$$

We prove that $\widehat{\mathbf{W}}-\mathbf{W}=(\widehat{\mathbf{V}}-\mathbf{V})+\mathbf{R}$ *with* $\|\mathbf{R}\|=\mathcal{O}(s)$ *when* s *is close to* 0*. Here* $\|\mathbf{R}\|=\mathcal{O}(s)$ *means that there exist positive numbers* M *and* δ *such that* $\|\mathbf{R}\|\le M\times s$ *when* $\|s\|<\delta$*.*

Proof. First, we know that when $\mathbf{X}_n$ is invertible and s is small enough, we have [12]

$$\begin{aligned}(\mathbf{X}_n+s\mathbf{I})^{-1}&=\mathbf{X}_n^{-1}-s\mathbf{X}_n^{-2}+s^2\mathbf{X}_n^{-3}-\cdots\\&=\mathbf{X}_n^{-1}+\mathcal{O}(s).\end{aligned}$$

This can be validated by multiplying $(\mathbf{X}_n+s\mathbf{I})$ on both sides of the equation and observing that both sides become identity matrices. With this property, we can reformulate $\widehat{\mathbf{W}}-\mathbf{W}$ as

$$\begin{aligned}\widehat{\mathbf{W}}-\mathbf{W}&=\Big(\frac{1}{N}\sum_n(\mathbf{X}_n+s\mathbf{I})\Big)^{-1}-\frac{1}{N}\sum_n(\mathbf{X}_n+s\mathbf{I})^{-1}\\&=\Big(\frac{1}{N}\sum_n\mathbf{X}_n+s\mathbf{I}\Big)^{-1}-\frac{1}{N}\sum_n(\mathbf{X}_n^{-1}+\mathcal{O}(s))\\&=(\widehat{\mathbf{V}}^{-1}+s\mathbf{I})^{-1}-\frac{1}{N}\sum_n\mathbf{X}_n^{-1}-\mathcal{O}(s)\\&=\widehat{\mathbf{V}}+\mathcal{O}(s)-\mathbf{V}-\mathcal{O}(s)\\&=(\widehat{\mathbf{V}}-\mathbf{V})+\mathcal{O}(s).\end{aligned}$$

□

So we have $\widehat{\mathbf{W}}-\mathbf{W}\approx\widehat{\mathbf{V}}-\mathbf{V}$ for small s. Now if we take $n=t$, $N=T$, $\mathbf{Y}_n=\mathbf{S}_{t|t-1}$, and $\mathbf{X}_n=\mathbf{S}_{t-1|t-1}$, then equation $\mathbf{Y}_n=\mathbf{X}_n+s\mathbf{I}$ above becomes (4). With these replacements, $\widehat{\mathbf{W}}-\mathbf{W}\approx\widehat{\mathbf{V}}-\mathbf{V}$ gives (A48). Note that $\mathbf{S}_{t|t}$ and $\mathbf{S}_{t|t-1}$ are periodic with period T. So (A48) holds regardless of the initial index in the summation (since the summation is over one period).

Appendix J: Solving the Discrete Riccati Equation (DRE) Analytically to Find the Average Estimation Covariances

Here we show how to solve for the average of the prediction and posterior covariances $\widetilde{\mathbf{S}}_-$ and $\widetilde{\mathbf{S}}_+$ in (A19) and (A20) for continuous valued observations in the KF. The same technique can be applied for solving these covariances $\widetilde{\mathbf{Q}}_-$ and $\widetilde{\mathbf{Q}}_+$ in (A36) and (A37) for point process observations in the PPF. In particular, we show that these average covariances can be obtained as solutions to the discrete Riccati equation (DRE) and derive an algorithm that can find this solution analytically for our case.

First, we consider the general DRE as follows [8]

$$\mathbf{X} = \mathbf{A}'\mathbf{X}\mathbf{A} + \mathbf{Q} - \mathbf{A}'\mathbf{X}\mathbf{B}(\mathbf{R} + \mathbf{B}'\mathbf{X}\mathbf{B})^{-1}\mathbf{B}'\mathbf{X}\mathbf{A}, \tag{A49}$$

where $\mathbf{X}$ is the unknown symmetric matrix, $\mathbf{A}$, $\mathbf{B}$, $\mathbf{Q}$ and $\mathbf{R}$ are known coefficient matrices, and $\mathbf{Q}$ and $\mathbf{R}$ are symmetric. The equivalent equation set is [8]

$$\begin{aligned} \mathbf{X} &= \mathbf{A}'\mathbf{Y}\mathbf{A} + \mathbf{Q} \\ \mathbf{Y}^{-1} &= \mathbf{X}^{-1} + \mathbf{B}\mathbf{R}^{-1}\mathbf{B}' \end{aligned} \tag{A50}$$

where $\mathbf{Y}$ is a dummy variable. We now see that equations (A19)–(A20) and equations (A36)–(A37) are a special form of (A50) with $\mathbf{A} = \mathbf{I}_n$, $\mathbf{Q} = q\mathbf{I}_n$ with $q > 0$, and $\mathbf{B}\mathbf{R}^{-1}\mathbf{B}' = \mathbf{H}$ where $\mathbf{H}$ is invertible. In (A20) $\mathbf{H} = \mathbf{H}_{ave} = \mathbf{U}\ diag(h_1, ..., h_n)\ \mathbf{U}'$ $(0 < h_i \leq h_{i+1})$ and in (A37) $\mathbf{H} = \mathbf{M}_{ave} = \mathbf{U}\ diag(a_1, ..., a_n)\ \mathbf{U}'$ $(a_i \leq a_{i+1})$. This special form can thus be written as

$$\mathbf{X} = \mathbf{Y} + \mathbf{Q} \tag{A51}$$

$$\mathbf{Y}^{-1} = \mathbf{X}^{-1} + \mathbf{H} \tag{A52}$$

Note that by taking $\mathbf{X} = \widetilde{\mathbf{S}}_-$ and $\mathbf{Y} = \widetilde{\mathbf{S}}_+$, equations (A19) and (A20) are equivalent to equations (A51) and (A52), respectively, with q playing the role of the learning rate. So if we solve for $\mathbf{X}$ and $\mathbf{Y}$, we have also solved for $\widetilde{\mathbf{S}}_-$ and $\widetilde{\mathbf{S}}_+$ in the KF. The same conclusion holds for $\widetilde{\mathbf{Q}}_-$ and $\widetilde{\mathbf{Q}}_+$ in the PPF.

In the following, we derive an algorithm that can solve for $\mathbf{X}$ and $\mathbf{Y}$ analytically for this special form. The solution of $\mathbf{Y}$ is given by the following lemma.

Lemma 3. *Denote the singular value decomposition (SVD) of* $\mathbf{Y}$ *and* $\mathbf{H}$ *in* (A52) *by*

$$\begin{aligned} \mathbf{Y} &= \mathbf{U}\mathbf{D}_Y\mathbf{U}' \ \ \textit{with}\ \ \mathbf{D}_Y = diag(y_1, ..., y_n)\ (y_i \geq y_{i+1} > 0) \\ \mathbf{H} &= \mathbf{V}\mathbf{D}_H\mathbf{V}' \ \ \textit{with}\ \ \mathbf{D}_H = diag(h_1, ..., h_n)\ (h_{i+1} \geq h_i > 0) \end{aligned}$$

then $\mathbf{U} = \mathbf{V}$ *and* $y_k = \frac{2}{\sqrt{h_k^2 + 4h_k q^{-1}} + h_k}$ *for* $\forall k \in \{1, \cdots, n\}$.

Proof. From (A51), $\mathbf{X} = \mathbf{U}(\mathbf{D}_Y + q\mathbf{I}_n)\mathbf{U}'$. Substituting this into (A52), we get $\mathbf{H} = \mathbf{V}\mathbf{D}_H\mathbf{V}' = \mathbf{Y}^{-1} - \mathbf{X}^{-1} = \mathbf{U}\mathbf{D}_\Gamma\mathbf{U}'$ where $\mathbf{D}_\Gamma = diag(\gamma_1, ..., \gamma_n)$ and $\gamma_i = \frac{1}{y_i} - \frac{1}{y_i + q}$. Because both sides are positive definite, they can be diagonalized simultaneously, i.e., $\mathbf{U} = \mathbf{V}$ and $\mathbf{D}_H = \mathbf{D}_\Gamma$, so $h_k = \gamma_k$. After solving this quadratic equation, we get $y_k = \frac{2}{\sqrt{h_k^2 + 4h_k q^{-1}} + h_k}$. □

In [13], a general solution is given for equations (A50). But it is hard to analyze the relation between q, the learning rate in our context, and matrix $\mathbf{Y}$ in that general solution. Indeed, to derive the calibration algorithm, we need to be able to write this relationship explicitly. Hence we derived the above algorithm to derive an explicit relationship and thus enable solving for the optimal learning rate. Having found $\mathbf{Y}$, we can now find $\mathbf{X}$ as $\mathbf{X} = \mathbf{Y} + \mathbf{Q}$. So we can solve for both $\mathbf{X}$ and $\mathbf{Y}$ as functions of $\mathbf{Q} = q\mathbf{I}_n$ and $\mathbf{H}$.

References

1. Gilja V, Nuyujukian P, Chestek CA, Cunningham JP, Yu BM, Fan JM, et al. A High-Performance Neural Prosthesis Enabled by Control Algorithm Design. Nat Neurosci. 2012 Dec;15:1752–1757.

2. Orsborn AL, Dangi S, Moorman HG, Carmena JM. Closed-Loop Decoder Adaptation on Intermediate Time-Scales Facilitates Rapid BMI Performance Improvements Independent of Decoder Initialization Conditions. IEEE Trans Neural Syst Rehabil Eng. 2012 Jul;20(4):468–477.

3. So K, Dangi S, Orsborn AL, Gastpar MC, Carmena JM. Subject-specific modulation of local field potential spectral power during brain-machine interface control in primates. J Neural Eng. 2014 Feb;11:026002.

4. Shanechi MM, Orsborn AL, Carmena JM. Robust Brain-Machine Interface Design Using Optimal Feedback Control Modeling and Adaptive Point Process Filtering. PLoS Comput Biol. 2016;12(4):e1004730.

5. Shanechi MM, Orsborn AL, Moorman H, Gowda S, Dangi S, Carmena JM. Rapid Control and Feedback Rates Enhance Neuroprosthetic Control. Nature Communications. 2017;(8):13825.

6. Eden UT, Frank LM, Barbieri R, Solo V, Brown EN. Dynamic analysis of neural encoding by point process adaptive filtering. Neural Comput. 2004;16:971–998.

7. Haykin SS. Adaptive filter theory. Pearson Education India; 2008.

8. Lancaster P, Rodman L. Algebraic riccati equations. Oxford University Press; 1995.

9. Bittanti S, Laub AJ, Willems JC. The Riccati Equation. Springer New York; 1991.

10. Bucy RS, Joseph PD. Filtering for stochastic processes with applications to guidance. vol. 326. American Mathematical Soc.; 1987.

11. Chen CT. Linear system theory and design. Oxford University Press, Inc.; 1995.

12. Petersen KB, Pedersen MS, et al. The matrix cookbook. Technical University of Denmark. 2008;7:15.

13. Vaughan D. A nonrecursive algebraic solution for the discrete Riccati equation. Automatic Control, IEEE Transactions on. 1970;15(5):597–599.
